# Regulation of charge carrier transportation in D–π–A type covalent organic frameworks for promoting photocatalytic H_2_O_2_ production

**DOI:** 10.1039/d5sc02875b

**Published:** 2025-08-08

**Authors:** Hailing Ma, Yangpeng Zhang, You Wu, Qianfeng Gu, Zhonghua Li, Qichun Zhang

**Affiliations:** a School of Chemistry and Chemical Engineering, Harbin Institute of Technology Harbin 150001 P. R. China lizh@hit.edu.cn; b Department of Materials Science and Engineering, City University of Hong Kong Hong Kong SAR 999077 P. R. China qiczhang@cityu.edu.hk

## Abstract

Covalent Organic Frameworks (COFs) with diverse conjugated structures are extensively utilized as promising photocatalysts for hydrogen peroxide (H_2_O_2_) production. However, the current applications are constrained by the rapid recombination of photogenerated carriers and the slow reaction kinetics. To address these issues, in this study, we design and prepare four COF photocatalysts with a distinct donor–π–acceptor (D–π–A) structure to regulate the photogenerated charge carrier transportation. Significantly, Tf-TAPT-COF containing triazine units shows the most elevated rate of H_2_O_2_ evolution. This is primarily attributed to the electron-withdrawing capacity of N centers in COFs following the order: triazine > pyrimidine > pyridine > benzene, leading to the enhanced transportation of electrons along the π-bridge from the donor to the acceptor. In the absence of a sacrificial agent, the photocatalytic H_2_O_2_ production of Tf-TAPT-COF can reach 2700 μmol g^−1^ h^−1^, surpassing most of the similar photocatalysts reported previously. A series of experimental characterization studies and theoretical calculations indicate that increasing the number of N atoms in the N-heterocycles of COFs can enhance the transportation of photogenerated carriers along the π-bridge. It enables COFs to facilitate the oxygen reduction reaction (ORR) *via* both π-bridges and acceptor dual sites, and the water oxidation reaction (WOR) occurs on the donor, thereby significantly improving their activity in photocatalytic H_2_O_2_ production. This research provides a promising approach for the regulation of charge carrier transportation in COFs and the performance improvement in full photosynthesis of H_2_O_2_ based on COFs.

## Abstract 2

The four D–π–A type COFs with different electron-withdrawing units (N-heterocycles) can regulate the transportation of photogenerated charge carriers.

## Graphical abstract

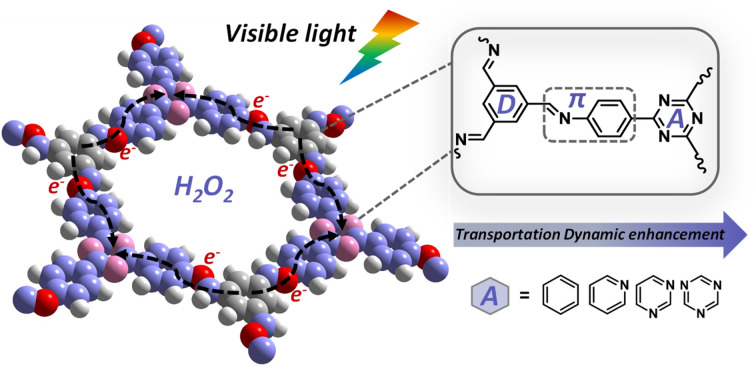

## Introduction

Hydrogen peroxide (H_2_O_2_) serves as a highly active and environmentally friendly oxidant with different applications in various industrial and health fields, including organic synthesis, wastewater treatment, and medical hygiene.^1,2^ The market demand for H_2_O_2_ is anticipated to grow significantly, with global annual production forecasted to reach 5.7 million metric tons by 2027.^3,4^ Currently, the anthraquinone process represents the predominant method for H_2_O_2_ production. Although this method facilitates mass production, it consumes significant amounts of energy and requires toxic organic materials and solvents, leading to severe environmental pollution.^5^ In recent years, the photocatalytic production of H_2_O_2_ has attracted wide attention. The photocatalytic production of H_2_O_2_, utilizing O_2_ and H_2_O as raw materials, solar energy as the energy source, and semiconductor materials as photocatalysts, represents the characteristics of sustainable chemical processes. This method offers advantages such as mild reaction conditions, simple and controllable operations, energy-saving and environmental protection.^6,7^ In this regard, the rational design and preparation of photocatalysts with high activity and selectivity are crucial for the efficient production of H_2_O_2_.

Graphite-like carbon nitride, titanium dioxide, and various semiconductor nanomaterial photocatalysts have gained considerable attention due to their straightforward preparation methods, low toxicity, and high chemical stability.^8–12^ However, these materials encounter several challenges such as poor utilization of visible light, wide band gaps, and susceptibility to photocorrosion.^13–15^ Beyond the traditional semiconductors that have been extensively researched in recent years, emerging materials such as organic frameworks with notable visible light absorption, tunable structures, and additional advantages are being thoroughly investigated and utilized.^16,17^ Compared to other crystalline porous materials, covalent organic frameworks (COFs) offer significant advantages, such as large specific surface areas, tunable pore sizes, and modifiable pore environments, making them highly promising candidates in the field of photocatalysis.^18,19^ The photocatalytic performance of COFs is currently enhanced primarily through structural regulation methods, such as molecular design and the introduction of guest species.^20,21^ Here are some primary strategies: (1) incorporating units with the capabilities to absorb broad-spectrum light can enhance the efficiency of light-harvesting. For example, metal–organic complexes or specific photosensitive groups can be integrated into COFs to expand their response range to cover visible and near-infrared light;^22,23^ (2) utilizing heterojunction structures through compositing COFs with semiconductor materials, which effectively promotes the separation of electron–hole pairs, reduces recombination rates, and enhances quantum efficiency;^24,25^ and (3) modifying the COF surface by grafting specific functional molecules or nanoparticles, which not only adjusts their hydrophilicity/hydrophobicity but also directly contributes to the photocatalytic reaction pathway, thereby enhancing photocatalytic activity.^26,27^ Furthermore, the development of COFs with extended photogenerated charge-separated states is pivotal in enhancing photocatalytic efficiency. This not only helps to reduce the recombination rate of photogenerated carriers, but also prolongs the existence time of active species to create more opportunities for the photocatalytic reaction.^28,29^

The D–π–A type COFs have shown significant potential in photoelectric conversion and photocatalysis applications.^30,31^ By ingeniously designing donors (D), acceptors (A), and the π-bridges that connect them, these structures aim to optimize charge separation, mobility, and transfer efficiency.^32^ The conjugated π-bridge facilitates rapid electron migration through the formation of a continuous electron cloud.^33^ However, traditional π-bridges primarily consist of large aromatic rings, such as benzene and naphthalene, whose broad transmission pathways and spatial twisting angles lead to slower carrier transportation rates.^34,35^ Gao *et al.* introduced building blocks with varying electron-withdrawing capabilities into COFs, thus enhancing intramolecular charge separation forces, and designed a novel vinylene-linked MCOF that achieved efficient photocatalytic CO_2_ reduction.^36^ Inspired by this work, H_2_O_2_ photocatalysts have concentrated on enhancing the electron affinity of acceptors within COFs. This enhancement aims to improve the transportation of photogenerated charges across the π-bridge, which significantly enhances the charge separation efficiency and photocatalytic performance.

In this work, we synthesized four distinct COF-based photocatalysts with a well-defined D–π–A architecture *via* Schiff base reactions. These materials were designated as Tf-TAPB-COF, Tf-TAPP-COF, Tf-TAPPM-COF, and Tf-TAPT-COF, respectively, incorporating benzene, pyridine, pyrimidine, and triazine structures. All four COFs were capable of photocatalytically generating H_2_O_2_ through dual channels involving the oxygen reduction reaction (ORR) and the water oxidation reaction (WOR), with ORR serving as the primary contributor. Both experimental results and theoretical calculations indicate that the number of N atoms in the N-heterocycles of COFs can enhance the electron-withdrawing ability of the acceptor and increase the transportation of photogenerated electrons, thereby effectively regulating the separation efficiency of photogenerated electron–hole pairs and significantly improving the photocatalytic activity. This makes the π-bridge (benzene beside N-heterocycles) as the main active site for the ORR, and the acceptor (N-heterocycle) as the secondary ORR active site, significantly improving the photocatalytic activity of COFs. Among them, Tf-TAPT-COF containing triazine units exhibits excellent photocatalytic activity, achieving H_2_O_2_ production rates of up to 2700 μmol g^−1^ h^−1^ under pure oxygen conditions and 1652 μmol g^−1^ h^−1^ under air conditions. This work provides significant research progress in structurally regulating COFs from an atomic level to enhance the transportation of photo-generated electrons, improving the utilization of solar energy.

## Results and discussion

### Synthesis and characterization of four COFs

**Fig. 1:**
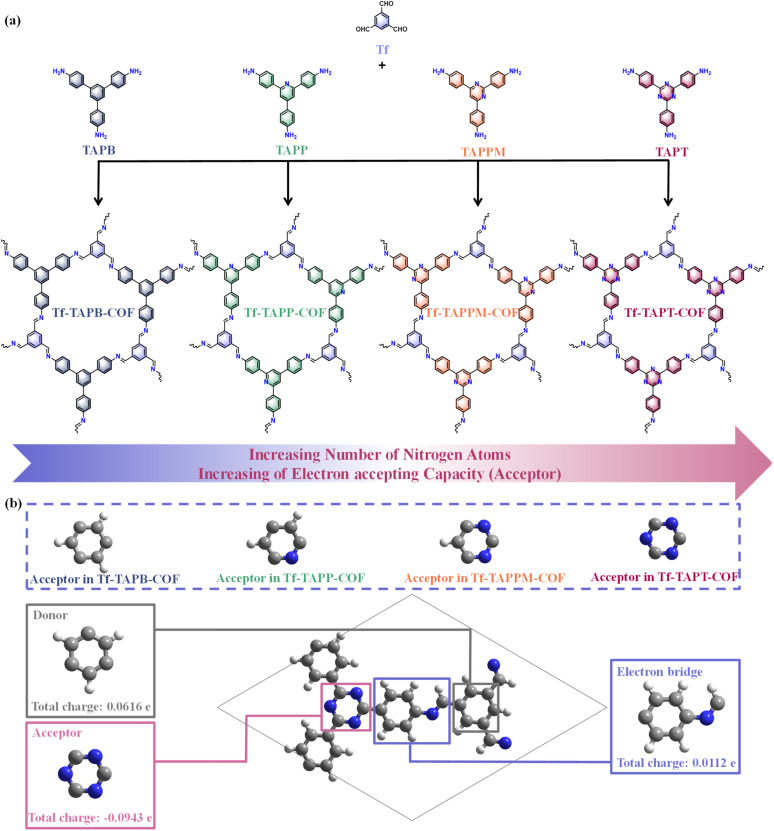
(a) Design and synthesis of Tf-TAPB-COF, Tf-TAPP-COF, Tf-TAPPM-COF, and Tf-TAPT-COF. (b) DFT calculation on Hirshfeld charge distribution of Tf-TAPT-COF, where gray, white, and blue spheres represent C, H, and N atoms, respectively.

Four COFs were solvothermally synthesized *via* Schiff-base condensation between the aldehyde monomer (Tf) and various triamines (TAPB, TAPP, TAPPM, and TAPT). Fig. 1a illustrates the design and synthesis of Tf-TAPB-COF, Tf-TAPP-COF, Tf-TAPPM-COF, and Tf-TAPT-COF. As shown in Fig. 1b, S1 and Tables S1–S4, the analysis of Hirshfeld charge distribution, based on Density Functional Theory (DFT) calculations, indicates that the amino monomers of Tf-TAPP-COF, Tf-TAPPM-COF, and Tf-TAPT-COF containing pyridine, pyrimidine, and triazine fragments (N-heterocycles), respectively, possess electron-withdrawing characteristics and can serve as electron acceptors. For Tf-TAPB-COF lacking N-heterocycles, the electron-withdrawing effect of the acceptor is weakened due to the electron-withdrawing nature of the imine bond, causing electrons to tend to concentrate at the position of the imine bond. Conversely, the aldehyde monomers of the four COFs have electron-rich benzene fragments that can serve as electron donors. There is a π-bridge (benzene beside N-heterocycles) between the donor and the acceptor, establishing a built-in electric field strong enough to facilitate rapid electron transfer.^37,38^ The transportation within the structure of all COFs is regulated by precisely engineering the acceptor structure and electron-withdrawing capability in the D–π–A configuration, which anticipates to suppress charge recombination and enhance the effective separation of excitons.

**Fig. 2:**
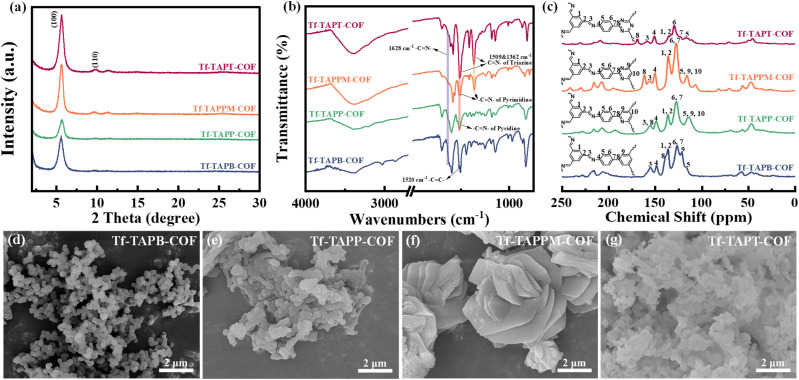
(a) Experimental powder X-ray diffraction (PXRD) patterns, (b) Fourier transform infrared (FT-IR) spectra, (c) solid-state ^13^C cross-polarization magic angle spinning NMR spectra, and (d–g) scanning electron microscopy (SEM) images of Tf-TAPB-COF, Tf-TAPP-COF, Tf-TAPPM-COF, and Tf-TAPT-COF.

The powder X-ray diffraction (PXRD) patterns revealed that the four COFs were highly crystalline polymers to minimize the impact of crystallinity on photocatalyst activity, as shown in Fig. 2a. The experimental PXRD patterns of the four COFs display a prominent peak at ≈5.60°, corresponding to the (100) plane, accompanied by a weak peak at ≈9.66°corresponding to the (110) plane. Structural models featuring eclipsed (AA) and staggered (AB) stacking are constructed using Materials Studio, and the results indicate that the experimental PXRD patterns are consistent with the simulation outcomes derived from the AA stacking model for the four COFs (Fig. S2). The four synthesized COFs were characterized using Fourier transform infrared spectroscopy (FT-IR) (Fig. 2b). The FT-IR spectra reveal a C=N stretching vibration at 1628 cm^−1^, indicative of the successful condensation reaction between –NH_2_ and –CHO groups. Additionally, the characteristic peaks of N-heterocycles in amino monomers are preserved in the final COFs. This observation aligns with the reported spectra of analogous COFs, suggesting the successful formation of the desired compounds.^39,40^ As shown in Fig. 2c, further characterization of the local chemical structure of these COFs was conducted using solid-state ^13^C cross-polarization magic angle spinning NMR (^13^C CP/MAS NMR). The resonance peak (peak 3) at 157 ppm of the four COFs confirms the presence of the C=N bond. Notably, the peak (peak 8) associated with the = C-Ar- functional group in the N-heterocycle experiences a downfield shift (towards higher chemical shift values) as the number of N increased. The values are recorded as 139.19, 155.64, 161.28, and 169.06 ppm for Tf-TAPB-COF, Tf-TAPP-COF, Tf-TAPM-COF, and Tf-TAPT-COF, respectively. This downfield shift was attributed to the enhanced electronegativity of sp^2^-hybridized nitrogen (sp^2^-N) atoms, leading to deshielding effects on the adjacent carbon atoms.^41^ These findings are in strong agreement with the FT-IR spectral data, confirming the successful synthesis of the four COFs.

As shown in Fig. S3, the thermal stability of the four COFs was evaluated *via* thermogravimetric analysis (TGA). The as-synthesized COFs exhibit outstanding thermal stability retaining their integrity up to 500 °C. Furthermore, the porous characteristics of the four COFs were evaluated using N_2_ adsorption–desorption isotherms (Fig. S4), revealing that they exhibit typical type-I isotherm characteristics. The Brunauer–Emmett–Teller (BET) surface areas for Tf-TAPB-COF, Tf-TAPP-COF, Tf-TAPPM-COF, and Tf-TAPT-COF are calculated to be 156.06, 251.85, 312.35, and 541.61 m^2^ g^−1^, respectively. This indicates that the number of N atoms in the N-heterocycle, based on the amino acid monomers, significantly affects the BET surface area of the four COFs. Among them, Tf-TAPT-COF exhibits a larger specific surface area, thereby providing enough active sites that promote O_2_ adsorption. The pore sizes, as determined using the nonlocal density functional theory (NLDFT), are found to be 1.21, 1.19, 1.18, and 1.16 nm for Tf-TAPB-COF, Tf-TAPP-COF, Tf-TAPPM-COF, Tf-TAPT-COF respectively. These results provide compelling evidence that the four COFs with desirable porosity have been successfully synthesized. As shown in Fig. 2d–g and S5, the morphologies of the four COFs were characterized by scanning electron microscopy (SEM). The findings reveal that Tf-TAPB-COF and Tf-TAPP-COF consist of uniformly distributed small spherical particles and irregular nanobars, respectively. Within Tf-TAPPM-COF, a stratified structure is discernible, which stacks to form a flower-like morphology, with dimensions extending up to several micrometers. Tf-TAPT-COF comprises a limited number of pieces and irregular spherical particles. In conjunction with its transmission electron microscopy (TEM) image, a hollow structure within the lamellar layer of Tf-TAPT-COF is observed, exposing additional catalytically active sites (Fig. S6a–c). The energy dispersive X-ray spectroscopy (EDS) mapping suggests that C and N elements were equably distributed in the COFs (Fig. S6d–f).

### Electronic band structure and photocatalytic H_2_O_2_ performance

**Fig. 3:**
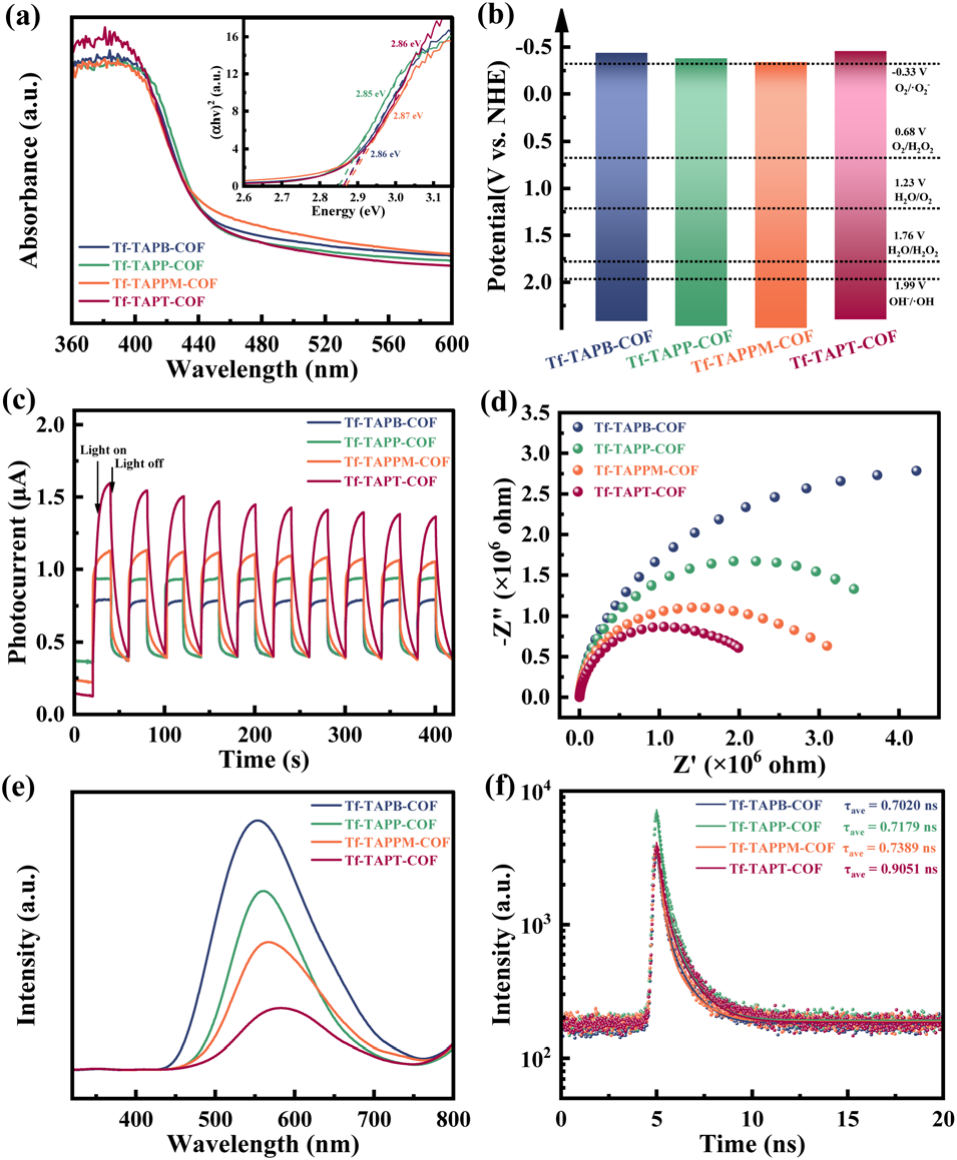
(a) The ultraviolet–visible diffuse reflection spectra (UV/Vis DRS), the inset shows the corresponding Tauc plots, (b) electronic band structures, (c) transient photocurrent response, (d) electrochemical impedance spectroscopy (EIS), (e) the steady-state photoluminescence (PL) measurements, and (f) time-resolved photoluminescence (TR-PL) spectra of Tf-TAPB-COF, Tf-TAPP-COF, Tf-TAPPM-COF, and Tf-TAPT-COF.

The ultraviolet-visible diffuse reflection spectroscopy (UV/Vis DRS) was employed to investigate the photophysical properties of the four COFs. As shown in Fig. 3a, the four COFs exhibited significant absorption within the visible light area. Based on the Kubelka–Munk function, the optical bandgaps of Tf-TAPB-COF, Tf-TAPP-COF, Tf-TAPPM-COF, and Tf-TAPT-COF were determined to be 2.86, 2.85, 2.87, and 2.86 eV, respectively. The semiconductor types and flat band positions (*V*_fb_) of the four COFs were determined through Mott–Schottky measurements (Fig. S7). The tangents of the longest straight-line segments of the four COFs have positive slopes, indicating that they are all n-type semiconductors.^42,43^ The conduction band (CB) potentials of Tf-TAPB-COF, Tf-TAPP-COF, Tf-TAPPM-COF, and Tf-TAPT-COF were calculated to be −0.44, −0.38, −0.34, and −0.46 eV (*vs.* NHE, pH = 7), respectively. Associated with these band gaps, the valence band (VB) potentials of Tf-TAPB-COF, Tf-TAPP-COF, Tf-TAPPM-COF, and Tf-TAPT-COF were 2.42, 2.47, 2.53, and 2.40 eV, respectively (Fig. 3b). Apparently, the band structures of the four COFs satisfy both the requirements for H_2_O_2_ synthesis from H_2_O (*E*_H_2_O2/H_2_O_ = +1.76 eV *vs.* NHE) and O_2_*E*_H_2_O_2_/˙O_2__^−^ = −0.33 eV *vs.* NHE), therefore, thermodynamic analysis indicates that the four COFs possess suitable band structures to make them effective photocatalysts for the overall process of H_2_O_2_ synthesis.^44,45^

The efficiency of electron-transfer at the interface for the four COFs was assessed utilizing transient photocurrent response and electrochemical impedance spectroscopy (EIS). Among them, Tf-TAPT-COF exhibits the strongest transient photocurrent response intensity and the smallest semicircle in its electrochemical impedance plot, indicating superior separation efficiency for photogenerated electrons and holes (Fig. 3c and d). Photoluminescence (PL) measurement was also performed to assess the interface charge separation efficiency. As shown in Fig. 3e, the emission peak of Tf-TAPT-COF is significantly lower than that of the other three COFs in the PL spectra. The reduced photoluminescence intensity indicates enhanced separation of photogenerated carriers, resulting in superior charge separation efficiency.^46^ Time-resolved photoluminescence (TR-PL) measurements were conducted to elucidate the charge transfer mechanisms (Fig. 3f). The excited-state absorption decay in Tf-TAPT-COF exhibits significantly longer average lifetime (0.9051 ns) compared to those in Tf-TAPB-COF (0.7020 ns), Tf-TAPP-COF (0.7179 ns), and Tf-TAPPM-COF (0.7389 ns). These results indicate that enhancing the electron-withdrawing ability of the acceptor facilitates charge carrier transportation, as evidenced by the substantially greater lifetime of Tf-TAPT-COF. The characterization analyses of electrochemical tests, PL spectroscopy, and TRPL spectroscopy conducted on all four COFs as previously described conclusively demonstrate that the photocatalytic H_2_O_2_ production capability of these materials adheres to the order: Tf-TAPT-COF > Tf-TAPPM-COF > Tf-TAPP-COF > Tf-TAPB-COF. Therefore, it can be speculated that the separation efficiency of photogenerated electron–hole pairs is affected by the charge transportation rate in COFs, and the separation efficiency of photogenerated electron–hole improves with the increase of acceptor electron-withdrawing strength. The most efficient electron transportation pathway is established in Tf-TAPT-COF containing the triazine structure, which ultimately enhances H_2_O_2_ photocatalytic production efficiency (discussed in detail below).

**Fig. 4:**
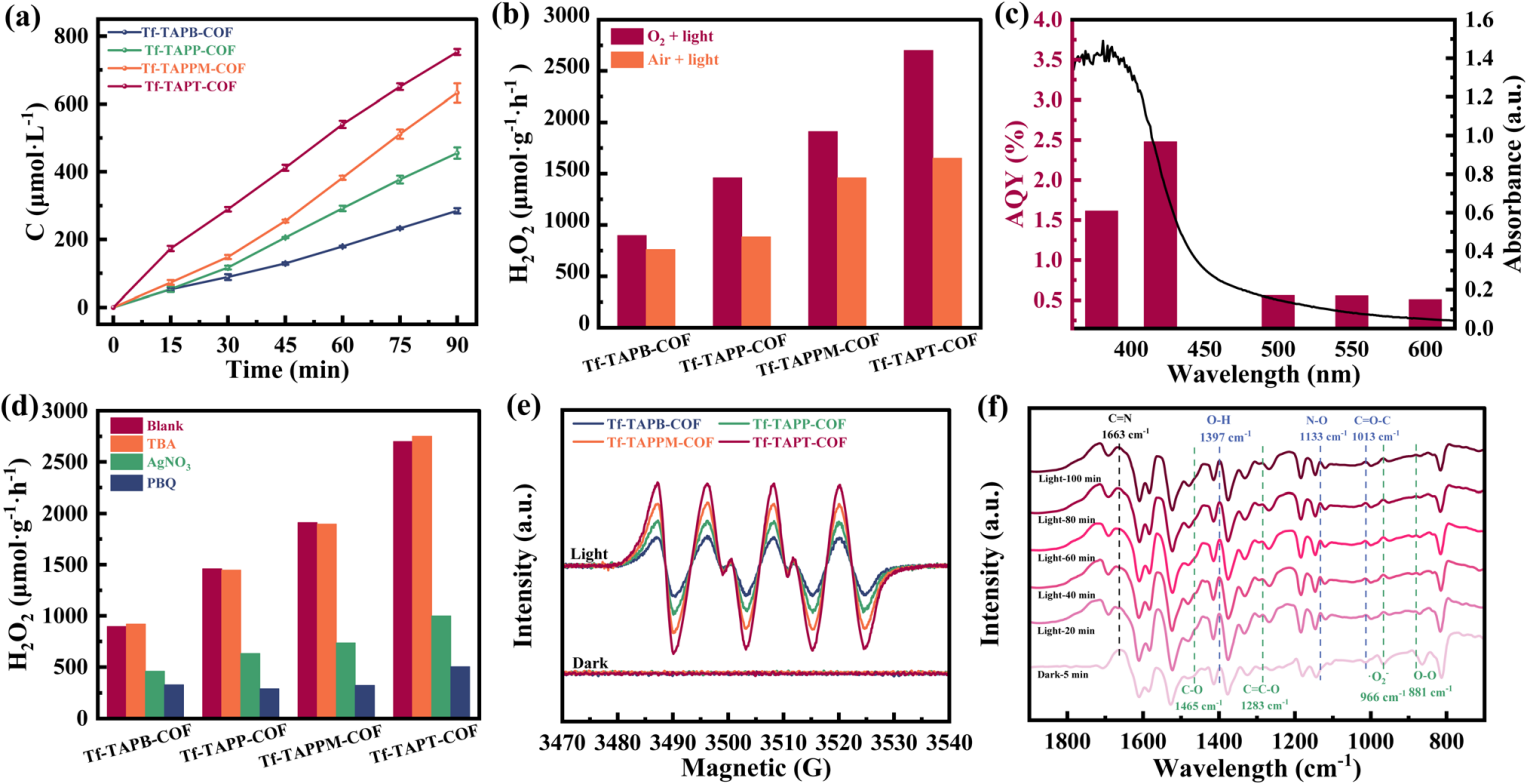
(a) Photocatalytic activities of Tf-TAPB-COF, Tf-TAPP-COF, Tf-TAPPM-COF, and Tf-TAPT-COF for H_2_O_2_ generation in deionized water without any sacrificial agents. (b) The control experiments of photocatalytic H_2_O_2_ production under different conditions (O_2_ and air). (c) Ultraviolet-visible diffuse reflection spectroscopy (UV/Vis DRS) spectrum and the apparent quantum yield (AQY) of Tf-TAPT-COF for H_2_O_2_ generation. (d) Amounts of H_2_O_2_ produced in *tert*-butanol (TBA, 10% v/v, as the ˙OH trapping agent), *p*-benzoquinone (PBQ, 5 mM, as the ˙O_2_^−^ trapping agent), and AgNO_3_ (10 mM, as the electron trapping agent) by Tf-TAPB-COF, Tf-TAPP-COF, Tf-TAPPM-COF, and Tf-TAPT-COF. (e) Electron paramagnetic resonance (EPR) spectra (DMPO – ˙O_2_^−^) of Tf-TAPB-COF, Tf-TAPP-COF, Tf-TAPPM-COF, and Tf-TAPT-COF under dark and light irradiation. (f) *In situ* diffuse reflectance infrared Fourier transform spectroscopy (DRIFTS) spectra *versus* illumination time for the photocatalytic system of Tf-TAPT-COF. The black dashed line represents the C=N in Tf-TAPT-COF, while the green and blue dashed lines indicate the intermediates in the stepwise 1e^−^ ORR pathway and the direct 2e^−^ WOR pathway during photocatalysis, respectively.

The photocatalytic performance of all four COFs for the generation of H_2_O_2_ was evaluated in deionized water and pure oxygen under visible-light irradiation (*λ* > 420 nm) without any sacrificial reagent. As shown in Fig. 4a and b, Tf-TAPT-COF displays a significantly higher H_2_O_2_ production rate (2700 μmol g^−1^ h^−1^) after 1 h, which is 3 times the rate for Tf-TAPB-COF (896 μmol g^−1^ h^−1^), 1.8 times the rate for Tf-TAPP-COF (1459 μmol g^−1^ h^−1^) and 1.4 times the rate for Tf-TAPPM-COF (1911 μmol g^−1^ h^−1^). All four COFs exhibit promising H_2_O_2_ generation rates, the descending order of their efficiencies is: Tf-TAPT-COF > Tf-TAPPM-COF > Tf-TAPP-COF > Tf-TAPB-COF. This trend correlates with the charge transportation efficiency within these COFs. These results indicate that regulating the transportation of electrons through the incorporation of N-heterocycles can effectively enhance the yield of photogenerated H_2_O_2_. Moreover, all four COFs retain high levels of H_2_O_2_ production under air conditions, with the photocatalytic H_2_O_2_ production rate of Tf-TAPT-COF reaching 1652 μmol g^−1^ h^−1^. Fig. 4c presents the apparent quantum efficiency (AQE) of Tf-TAPT-COF, which peaked at approximately 2.48% at 420 nm. The photocatalytic H_2_O_2_ production performance of Tf-TAPT-COF surpasses that of traditional photocatalysts and the majority of those recently reported in the field (Fig. S8 and Table S5).^47,48^ Apart from the excellent H_2_O_2_ production activity, the four COFs also exhibit reasonable photocatalytic reusability in four consecutive cycles, as shown in Fig. S9. Furthermore, the photocatalytic decomposition experiments on H_2_O_2_ indicate that the four COFs barely degrade H_2_O_2_, with its concentration remaining above 98% of the initial value under continuous visible-light irradiation for 1.5 h (Fig. S10). Comparing with the fresh samples, the PXRD patterns and FT-IR spectra of the four COFs after the photoreaction do not change significantly, suggesting that both their crystallinity and chemical structure are still well preserved (Fig. S11 and S12). The morphology of the four COFs after the photoreaction was characterized using SEM (Fig. S13 and S14), demonstrating that there are no substantial alterations in their morphology. These analyses suggest that the four COFs are resistant to light corrosion and serve as highly stable and effective photocatalysts.

### Photocatalytic H_2_O_2_ mechanism

In order to investigate the overall reaction of photocatalytic H_2_O_2_ production, a series of controlled experiments were carried out on four COFs. As shown in Fig. S15, in the absence of both catalyst and light, no H_2_O_2_ was detected, indicating that light and catalysts were essential conditions for the photosynthesis of H_2_O_2_; however, a small amount of H_2_O_2_ was generated under light and Ar conditions, indicating that the oxygen reduction reaction (ORR) is not the only pathway for H_2_O_2_ production, as the water oxidation reaction (WOR) also contributes. The trapping experiments delineated the reaction pathways of photosynthetic H_2_O_2_, identifying the ORR half-reactions and WOR half-reactions.^49,50^ To dissect the active intermediates and their pathways, as shown in Fig. 4d, *p*-benzoquinone (*p*BQ) and AgNO_3_ were used as scavengers for superoxide radicals (˙O_2_^−^) and photo-generated electrons (e^−^), respectively. The H_2_O_2_ yield was significantly reduced, thereby confirming the crucial role of ˙O_2_^−^ in the ORR and the central role of e^−^ in H_2_O_2_ formation, and suggesting a stepwise 1e^−^ ORR pathway.^51^ To further reveal the ORR mechanism of the four COFs, electron paramagnetic resonance (EPR) spectroscopy was performed using 5,5-dimethyl-1-pyhuntbjhrroline *N*-oxide (DMPO) as a radical spin trapping agent, and it was found that under visible-light conditions, typical characteristic signals of DMPO – ˙O_2_^−^ could be clearly monitored in all four COFs, while no such signals existed under dark conditions (Fig. 4e), indicating that ˙O_2_^−^ intermediates were generated.^52,53^ When *tert*-butanol (TBA) was added to the reaction system, the amount of generated H_2_O_2_ does not decrease (Fig. 4d), indicating that no ˙OH radicals were produced during the photocatalytic H_2_O_2_ production, thus ruling out the possibility of H_2_O_2_ production *via* a direct 2e^−^ WOR pathway.^54^ Furthermore, utilizing *in situ* diffuse reflectance infrared Fourier transform spectroscopy (DRIFTS) measurements, we investigated the photocatalytic mechanism of Tf-TAPT-COF, as shown in Fig. 4f. During the initial dark equilibration period with a continuous supply of O_2_, the characteristic band at 1663 cm^−1^ for Tf-TAPT-COF is indicative of C=N. Under visible-light irradiation, the vibration peaks of 1465 cm^−1^ (C–O) and 1283 cm^−1^ (C=C=O) in the *in situ* DRIFTS spectra of Tf-TAPT-COF gradually increase with the prolongation of reaction duration, suggesting that O_2_ is adsorbed onto C atoms of aniline and triazine. Then, vibrations at 881 cm^−1^ (O–O) and 966 cm^−1^ (˙O_2_^−^) are also evident in the *in situ* DRlFT spectra of Tf-TAPT-COF, indicating that the adsorbed O_2_ is reduced by electrons to form ˙O_2_^−^.^55^ Moreover, characteristic peaks at 1133 cm^−1^ (N–O) and 1397 cm^−1^ (O–H) were observed under visible-light conditions, suggesting significant molecular vibrations during photosynthesis of H_2_O_2_.^56^ These observations are consistent with a mechanism for the photosynthetic production of H_2_O_2_ in Tf-TAPT-COF that involves the coupling of stepwise 1e^−^ ORR and direct 2e^−^ WOR, as shown by previous results.

### Theoretical calculations

The intramolecular electron transfer in the four COFs was investigated using time-dependent density functional theory (TDDFT), demonstrating that they have prolonged charge-separated state (CSS) lifetimes. As shown in Fig. S16–S19, the calculated electronic excitations of Tf-TAPB-COF, Tf-TAPP-COF and Tf-TAPT-COF were mainly composed of two electronic excitation states of S0 → S7 and S0 → S8, which were contributed by the electronic transitions (HOMO−2 → LUMO) and (HOMO−2 → LUMO+1), electronic transitions (HOMO−2 → LUMO+1) and (HOMO−1 → LUMO+2), and electronic transitions (HOMO → LUMO+2) and (HOMO−1 → LUMO+2), respectively. The calculated electronic excitation of Tf-TAPPM-COF was mainly composed of the electronic excitation states of S0 → S7 and S0 → S10, which were contributed by electronic transitions (HOMO → LUMO+2) and (HOMO−1 → LUMO+2). The above calculations show that the four COFs have a good hole/electron distribution (Table S6).^57^ Based on DFT calculations, the *S*_m_ index (parameter denoting the hole–electron recombination degree, the smaller the better) of the four COFs in the excited state follows the order of Tf-TAPT-COF (0.471) < Tf-TAPB-COF (0.487) < Tf-TAPP-COF (0.493) < Tf-TAPPM-COF (0.538) (Table S7). It was observed that the electrons and holes in Tf-TAPT-COF exhibited greater resistance to recombination compared to those in the other three COFs. The impact of the electron-withdrawing characteristics of N-heterocycles on the efficiency of photogenerated charge transportation within systems can be assessed by integrating the intrinsic properties of D–π–A structures in the four COFs (Fig. 1b, S1 and Tables S1–S4). Based on the analysis of Hirshfeld charge distribution, Tf-TAPT-COF has the highest H_2_O_2_ production rate, while Tf-TAPB-COF has the lowest H_2_O_2_ production rate, which is mainly attributed to the fact that the electron-withdrawing ability of triazine (−0.0943 e) > pyrimidine (−0.0573 e) > pyridine (−0.0283 e) > benzene (−0.0062 e) promotes the transportation efficiency of photogenerated e^−^ from the donor to the acceptor.^58,59^ The charge density difference (CDD) between the relaxed excited state and the ground state provides an intuitive demonstration of electron redistribution. It highlights areas with increased electron density in the excited state relative to the ground state, thus providing a more accurate depiction of real-world conditions (Fig. S20).^60^ The results of CDD for all four COFs indicate that, from Tf-TAPB-COF to Tf-TAPT-COF, there is a trend of photogenerated charge transfer from the donor through the π-bridge toward the acceptor with enhancing acceptor electron-withdrawing ability of the COFs. With the increase of the number of N atoms in the N-heterocycle, the separation efficiency of photogenerated charges and holes effectively increases, which illustrates that the factor has a significant effect on the photocatalytic activity. Tf-TAPT-COF exhibits the strongest acceptor electron-withdrawing ability to transport more electrons, so that more photogenerated charges are concentrated on the π-bridge and transferred to triazine, and the dual sites act as the photoreduction part in the stepwise 1e^−^ ORR pathway; the photogenerated holes are mainly located in the benzene of the aldehyde monomer as the photooxidation part in the direct 2e^−^ WOR pathway. These findings indicate that Tf-TAPT-COF achieves a higher degree of photogenerated electron–hole pair separation, showcasing its superior photocatalytic performance. As shown in Fig. S21, the Gibbs free energy (Δ*G*) values of the four COFs indicate that there is no significant correlation between the Δ*G* trend of the rate-determining step (*O_2_ + H^+^ + e^−^ → *OOH) and performance, suggesting that thermodynamics does not play a dominant role in these reaction systems.

**Fig. 5:**
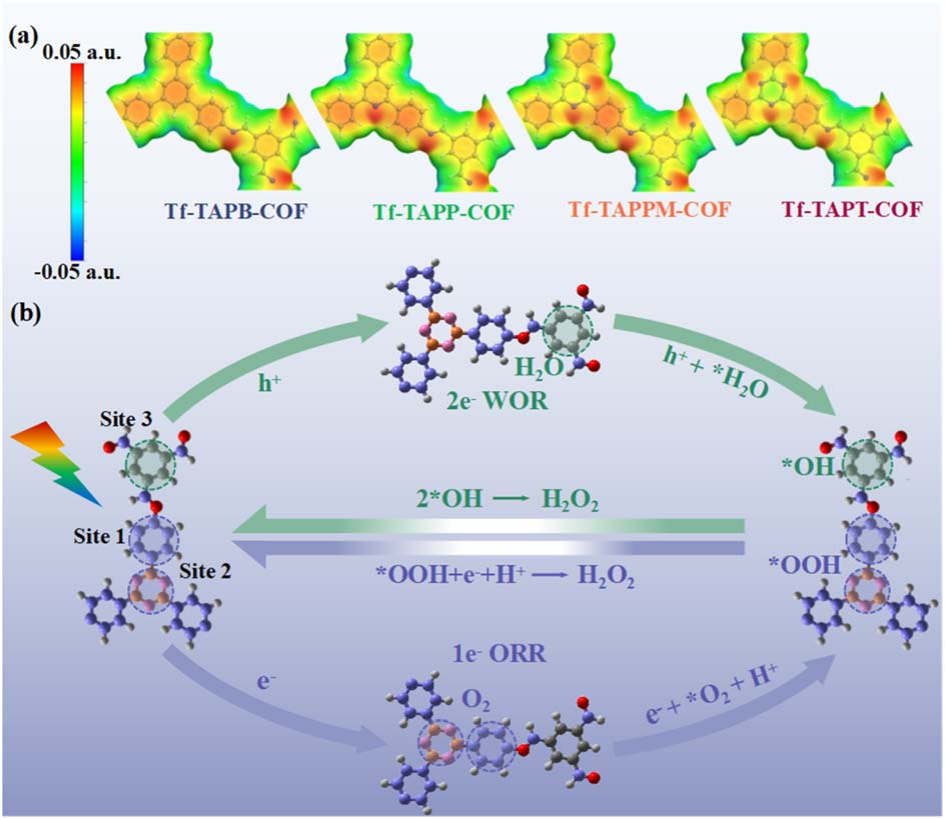
(a) Optimized geometrical structures and the electrostatic potential surfaces for model systems of Tf-TAPB-COF, Tf-TAPP-COF, Tf-TAPPM-COF, and Tf-TAPT-COF. (b) Proposed reaction mechanisms of photocatalytic H_2_O_2_ production for Tf-TAPT-COF. The grey, blue, and white spheres refer to C, N, and H, respectively.

In previous studies, photocatalysts with high polarity have demonstrated good separation efficiency of photogenerated charges and holes, thereby exhibiting excellent photocatalytic activity.^61–63^ Interestingly, the four COFs in this study exhibited the opposite trend: namely, the systems with lower polarity, showed the increased photocatalytic yield of H_2_O_2_. Essentially, the non-uniformity of charge distribution within a system manifests as molecular polarity. The electrostatic potential, dictated by this charge distribution, can directly access the polarity of the system through the characteristics of the molecular surface electrostatic potential distribution, known as the molecular polarity index (MPI) (larger MPI values indicate higher polarity).^64^ Among the four COFs, the molecular polarity index shows the trend of Tf-TAPB-COF (9.37 kcal mol^−1^) > Tf-TAPP-COF (9.32 kcal mol^−1^) > Tf-TAPPM-COF (8.94 kcal mol^−1^) > Tf-TAPT-COF (8.23 kcal mol^−1^), and the molecular polarity surface area also displays the same trend (Table S7). The uneven charge distribution in Tf-TAPB-COF is attributed to the electron-withdrawing effect of C=N, which results in high polarization. On the other hand, in Tf-TAPP-COF, Tf-TAPPM-COF and Tf-TAPT-COF, the simultaneous presence of both C=N and N-heterocycles with electron-withdrawing groups, contributes to a more uniform charge distribution within the photocatalyst as a whole, thus reducing the molecular polarity, which again indicates that the enhancement of the electron-withdrawing ability of the acceptor in COFs can effectively enhance the photogenerated charge mobility. Based on the electrostatic potential surface, further analysis reveals that although Tf-TAPT-COF exhibits lower polarity, due to the strong electron-withdrawing ability of the triazine, C=N on the π-bridge and triazine are the main regions for generating photoexcited carriers (Fig. 5a).

The experimental and theoretical calculation results propose a possible mechanism for the photosynthesis of H_2_O_2_ in the four COFs (Fig. 5b). Under visible-light irradiation, the photocatalysts absorb sufficient energy to make photogenerated electrons (e^−^) transfer from benzene in aldehyde monomers through aniline (π-bridge) to the N-heterocycle, during which process, photo-generated charges are gradually accumulated on the π-bridge, becoming the primary active sites for the ORR, while some electrons transfer to the acceptor, making triazine units the secondary active sites for the ORR. There are holes (h^+^) left on the donor, which become the primary active sites for the WOR. Subsequently, O_2_ is reduced by e^−^ to generate reactive intermediates (˙O_2_^−^) and then interacts with protons to produce H_2_O_2_. In parallel, H_2_O is oxidized by holes (h^+^), resulting in the formation of H_2_O_2_ through a stepwise 1e^−^ ORR and direct 2e^−^ WOR overall reaction.

## Conclusions

In summary, we have proposed a simple and effective method to regulate the charge transportation in D–π–A type COFs. These COFs demonstrate exceptional charge separation and transfer efficiencies upon photoexcitation, alongside superior photocatalytic activity in H_2_O_2_ production. In particular, Tf-TAPT-COF displays high H_2_O_2_ production of 2700 μmol g^−1^ h^−1^ without any sacrificial agents due to the strong electron-withdrawing ability of triazine units. This factor indicates that Tf-TAPT-COF has superior photogenerated charge transportation. The present result that the rapid transfer of photogenerated charges can be realized through integrating N-heterocycles with varying numbers of N atoms in COFs provides a new strategy to design and prepare innovative photocatalysts for H_2_O_2_ production.

## Author contributions

Hailing Ma: conceptualization, data curation, formal analysis, investigation, methodology, validation, writing – original draft & editing; Yangpeng Zhang: data curation, formal analysis, investigation, methodology; You Wu: data curation, formal analysis, investigation, methodology; Qianfeng Gu: writing – original draft & editing; Zhonghua Li: conceptualization, investigation, funding acquisition, project administration, resources, supervision, writing – review & editing; Qichun Zhang: writing – original draft, project administration, writing – review & editing.

## Conflicts of interest

There are no conflicts to declare.

## Supplementary Material

SC-016-D5SC02875B-s001

## Data Availability

The data that support the findings of this study are available from the corresponding author upon reasonable request. Experimental materials, methods, detailed synthesis procedures, structural and morphological characterization, TGA, stability testing, mechanistic experiments, theoretical calculations. See DOI: https://doi.org/10.1039/d5sc02875b.
