# Supplementary material for: Regulation of charge carrier transportation in D–π–A type covalent organic frameworks for promoting photocatalytic H2O2 production
Source: Chem Sci. 2025 Aug 8;16(36):16668–77. doi: 10.1039/d5sc02875b (PMC12360219; doi:10.1039/d5sc02875b)
Supplement: SC-016-D5SC02875B-s001 [file SC-016-D5SC02875B-s001.pdf]

Supplementary Information (SI) for Chemical Science.
This journal is © The Royal Society of Chemistry 2025

Supplementary Information

Regulation of Charge Carrier Transportation in D-π-A Type Covalent Organic Frameworks for Promoting Photocatalytic H_2O_2 Production

Hailing Ma,[‡a] Yangpeng Zhang,[‡a] You Wu,[‡a] Qiangfeng Gu, [b] Zhonghua Li,*[a] and Qichun Zhang*[b]

[a.]School of Chemistry and Chemical Engineering, Harbin Institute of Technology, No.92, West Da-Zhi Street, Harbin, 150001, P. R. China

[b.]Department of Materials Science and Engineering, City University of Hong Kong, P. R. China

1. Materials and Methods

Materials. All solvents and reagents obtained from commercial sources were used without further purification. Mesitylene (99%), 1,4-dioxane (99%), ethanol (99.5%), tetrahydrofuran (99.5%), acetic acid (99.5%), hydrazine hydrate and 30% H_2O_2 stock solution were purchased from Aladdin. 4,4',4''-(1,3,5-Triazine-2,4,6-triyl)Trianiline (TAPT), 4,4',4''-(Pyrimidine-2,4,6-triyl)trianiline (TAPPM), 1,3,5-Tris(4-aminophenyl) benzene (TAPB), 1,3,5-Benzenetricarbaldehyd (Tf), 4-Nitrobezaldehyde, 4-Nitroacetophenone, N,N-diethyl-1,4-phenylene diamine sulfate (DPD, 97%) and peroxidase (POD, horseradish), ammonium acetate, $FeCl_3 \cdot 6H_2O$ and activated carbon were purchased from Macklin. Other chemical reagents were purchased from local chemical suppliers.

Characterizations and instruments. The composition, structure, and texture properties of the materials were investigated by powder X-ray diffraction (PXRD) patterns (X'Pert PRO, ANalytical), Fourier transform infrared (FT-IR) spectra (Nicolet is50, Thermo Fisher), scanning electron microscopy (SEM) micrographs (Hitachi SU8010), transmission electron microscopy (TEM) experiment (Tecnai G2F30), and thermogravimetric analyses (TGA) (TG Q50). N_2 adsorption-desorption isotherms were performed on a nitrogen adsorption analyzer (Atoso3rb-IQ2-MP) at 77 K. The pore size distributions were measured by the Non-Local Densitj,y Functional Theory (NLDFT) method. Optical properties were also studied by diffuse reflectance UV-vis spectroscopy (U-4100) with $BaSO_4$ as a reference sample in the wavelength range of 200-800 nm. The photoluminescence (PL) spectra of the samples were measured with a Bruker E580 spectrofluorometer at an excitation wavelength of 380 nm. Electron paramagnetic resonance (EPR) measurements were performed using a Bruker model A300 spectrometer. The electrochemical impedance spectra (EIS), Mott-Schottky plot, photocurrent-time profiles (i-t curves), and cyclic voltammetry measurements (CV) were recorded on the CHI660E electrochemical workstation with a standard three-electrode system with the photocatalyst-coated ITO as the working electrode, the Pt plate as the counter electrode, and a saturated Ag/AgCl electrode as a reference electrode. During the measurement, a 300 W Xenon lamp with a 420 nm cut-off filter was used as the light source. Na_2SO_4 (0.5 M) solution was used as the electrolyte. The as-synthesized COFs (5 mg) were added into a 1 mL methanol and 50 μL Nafion mixed solution, and the working electrodes were prepared by dropping the suspension onto an ITO glass substrate electrode surface and drying at room temperature.

Photocatalytic reactions. In a typical experiment, 10 mg photocatalysts and 50 mL deionized water were put in 100 mL photoreactor. The suspension was well dispersed by ultrasonication for 5 min and O_2 was bubbled into the suspension for 30 min in the dark. Prior to the photocatalytic tests, the suspension was stirred for 30 min in dark to reach the absorption and desorption equilibrium. Then, the system was irradiated with a Xenon lamp (CEL HXF300). After sampling every 15 min, the H_2O_2 content in the solution was detected after the catalyst was filtered. In order to explore the influence of different gases (Ar and Air) on the photocatalytic H_2O_2 generation activity, Ar was continuously injected into the reaction solution under dark conditions for 30 minutes to ensure that there was no residual O_2. After that, the photocatalysis test was carried out under the condition of continuous Ar flow. When reacting in air conditions, the reaction was carried out in open conditions, stirred in dark conditions for 30 minutes, and then the photocatalytic test was carried out. In order to explore the influence of different sacrificial agents on the H_2O_2 generation activity under the same conditions, the concentration of $AgNO_3$ and PBQ added to the reaction system were 10 mM and 5 mM, and the concentration of TBA added to the reaction system was 10%.

Determination of H_2O_2 concentration. The concentration of H_2O_2 was determined by POD method.[1] K_2HPO_4 (1.4378 g) and KH_2PO_4 (5.9675 g) were ultrasonically dissolved in 100 mL deionized water, and phosphate buffer solution (PBS) with pH = 7 was prepared in volumetric bottle to adjust pH when the concentration was measured. 0.1 g N, N-diethyl-1,4-phenylene diamine (DPD) sulfate was dissolved in 0.05 M H_2SO_4 solution (10 mg) as organic stabilizer and peroxidase (POD) (10 mg) was dissolved in 10 mL deionized water in order to detect color changes occurring in hydrogen peroxide reactions. When measuring concentration, 1.12 mL deionized water, 0.05

mL DPD solution, 0.4 mL 1 M PBS solution and 0.05 mL POD were taken from the liquid gun and then 1 mL photoreacted solution was added to the mixed solution, which was slowly shaken for 30 s to make the solution evenly mixed. H_2O_2 molecules reacted with peroxidase (POD) to produce a color change (with strong absorption near 551 nm). The absorbance of the mixture solution at 551 nm was measured by ultraviolet spectrophotometer (UH5300), and then the amount of H_2O_2 generated by each reaction could be calculated according to the standard curve.

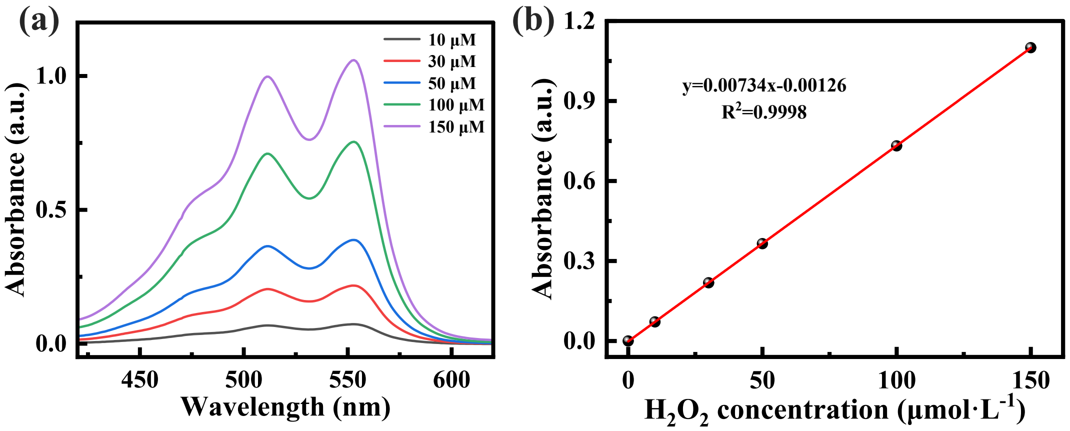

Apparent quantum efficiency (AQY) measurements. The AQY was determined by illuminating the sample with a 300 W Xe lamp at different bandpass values of 380 nm, 420 nm, 500 nm, 550 nm, and 600 nm. Prior to the photocatalytic reaction, the photocatalyst (25 mg) was added to 50 mL of deionized water and the mixture was then ultrasonicated and bubbled. The active area of the reactor was about 8.04 cm^2. Use PL-MW2000 optical radiometer to take the average value of monochromatic light intensity at five representative points. Therefore, the light intensity at 420 nm was calculated as 25.74 mW cm^{-2}. The AQY was calculated by the following formula：

$$\mathrm{AQY} = \frac{2 \times \mathrm{H_2O_2}\ \text{formed (mol)}}{\text{the number of incident photons (mol)}} \times 100\%$$

DFT calculations. Time-dependent density functional theory (TD-DFT) calculations and the Hirshfeld charge distribution of the models were conducted employing the QuickStep code within CP2K 9.1. The PerdewBurke-Ernzehof (PBE) exchange-correlation functional was utilized in conjunction with the DFT-D3 (BJ) van der Waals correction. A vacuum region of 20 Å was used to eliminate the interaction of layers for 2D imine-linked COFs. A diagonalization method was adopted for these computations. Due to the extensive cell parameters of the COFs, calculations were executed at the Γ-point across the irreducible Brillouin zone. In this study, the DZVP-MOLOPT-SR-GTH basis sets were used with plane-wave and relative energy cutoffs of 400 Ry and 50 Ry, and then the excited state data were analyzed and partial graphs were drawn using Multiwfn 3.7 (dev) code.[2,3]

2. Synthetic Procedures

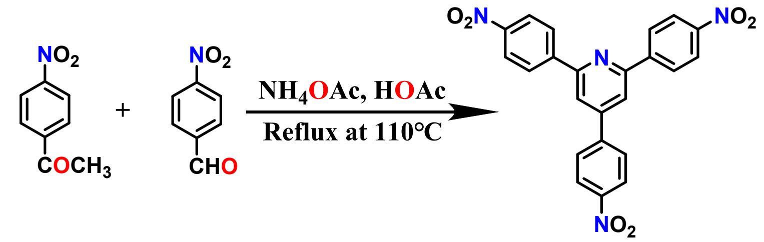

Scheme S1. Synthesis of 2,4,6-Tris(4-nitrophenyl)pyridine (TAPP-3NO_2).

Synthesis of 2,4,6-Tris(4-nitrophenyl)pyridine (TAPP-3NO_2): The amine building block was prepared by following the reported two step procedure.[4,5] In the first step, 10 mmol of 4-Nitrobezaldehyde and 20 mmol of 4-Nitroacetophenone were dissolved in 25 mL of acetic acid. Then 10 g of ammonium acetate was added to this mixture and refluxed at 110 °C for 3 h. Finally the deep orange precipitate was filtered and washed with acetic acid and cold Ethanol. The crude product was taken to the next step without any further purification.

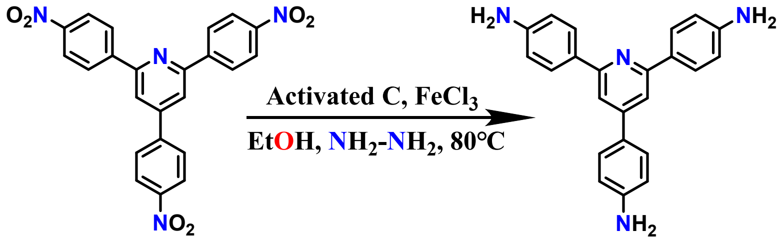

Scheme S2. Synthesis of 4,4',4''-(Pyridine-2,4,6-triyl)trianiline (TAPP).

Synthesis of 4,4',4''-(Pyridine-2,4,6-triyl)trianiline (TAPP): 1.5 g of 2,4,6-tris (4-nitrophenyl)pyridine was dissolved in 20 mL EtOH. About 60 mg of $FeCl_3$ and 200 mg of activated carbon were added to this solution and heated under reflux conditions for 30 min. To this, 4 mL of hydrazine hydrate in 4 mL EtOH was added and the mixture was refluxed for 12 h. The mixture was filtered as hot and the filtrate was poured in distilled water. A yellow precipitate appeared, which was filtered, washed with water and dried at 60 °C overnight. ^{1}H NMR spectrum of TAPP presented new signal peaks at δ = 5.38 (s, 4H) and 5.45 (m, 2H) due to the amino protons. The other fourteen protons of phenyl and pyridine ring overlapped in the range of δ = 6.64 - 7.96.

The corresponding ^{1}H NMR spectra was shown below:

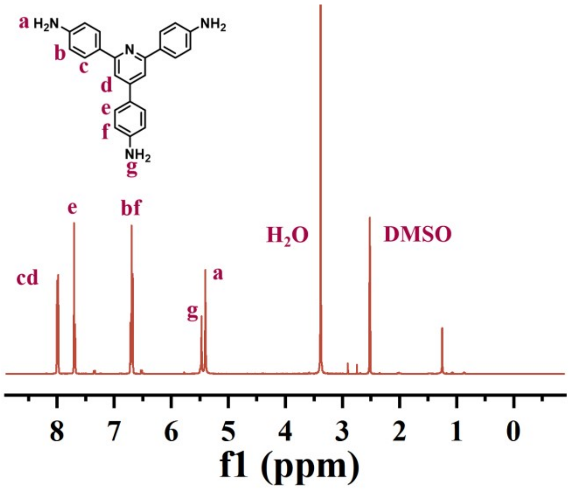

^{1}H NMR spectra of TAPP.

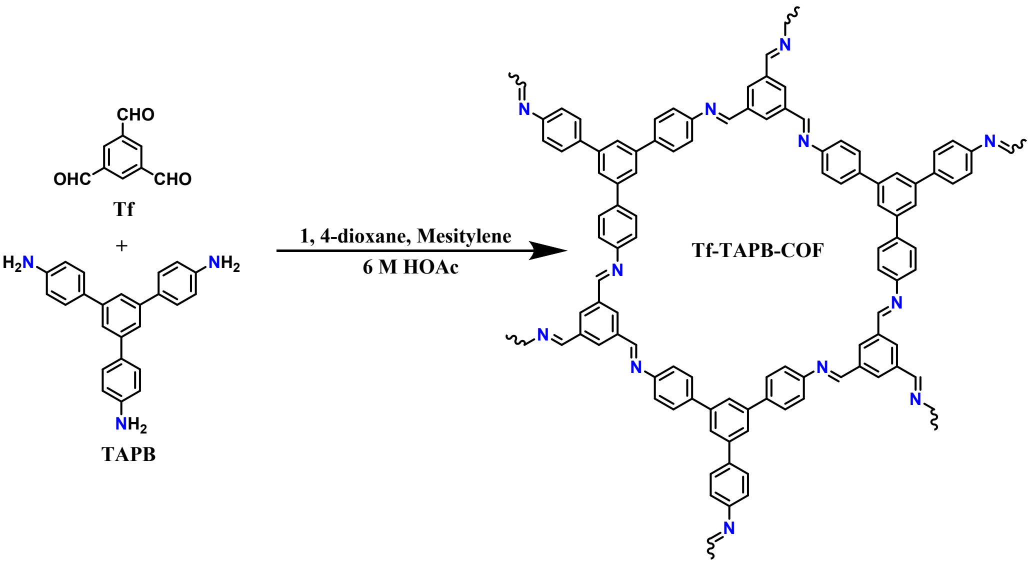

Scheme S3. Synthesis of the Tf-TAPB-COF.

Synthesis of the Tf-TAPB-COF: The Tf-TAPB-COF was synthesized according to a previously published procedure with a slight modification.[6] Tf (16.2 mg, 0.1 mmol), TAPB (35.1 mg, 0.1 mmol), mesitylene (2.25 mL) and 1, 4-dioxane (0.75 mL) were added into a Pyrex tube measuring 19 × 65 mm (o.d × length). Then, the tube was ultrasonicated for 10 min to disperse homogeneously as well and 6 M aqueous acetic acid (0.4 mL) was added the tube. The tube was flash frozen at 77 K (liquid N_2 bath) and degassed by three freeze-pump-thaw cycles and sealed off. The mixture was heated at 120 ºC and left undisturbed for 72 h. A yellow precipitate was isolated by filtration in Buchner funnel and was washed with THF until the filtrate was colorless. The obtained solid was transferred to a Soxhlet extractor and washed with THF for 24 h. Finally, the product was evacuated at 60 ºC under dynamic vacuum overnight to yield a faint yellow powder (yield, 82%).

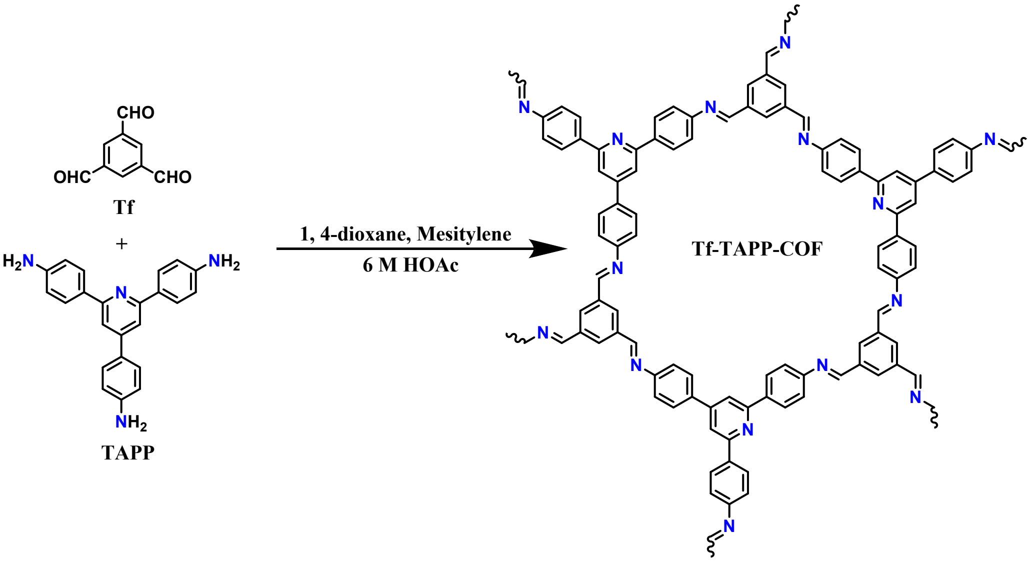

Scheme S4. Synthesis of the Tf-TAPP-COF.

Synthesis of the Tf-TAPP-COF: The synthesis procedure of the Tf-TAPP-COF was completely same with that of Tf-TAPB-COF, besides of using TAPP (35.2 mg, 0.1 mmol) as a substrate. Finally, an orange powder was obtained with a 75% yield.

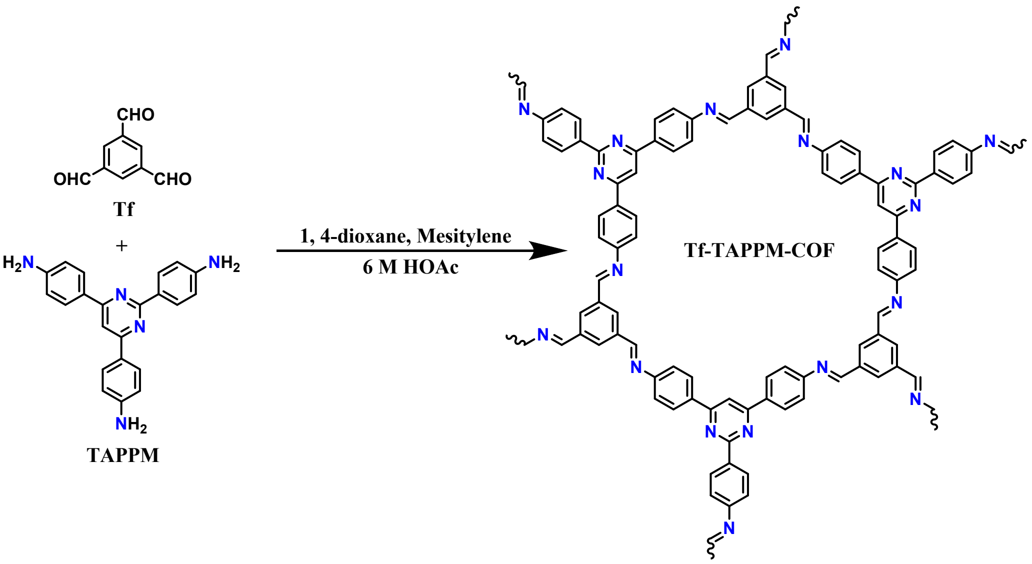

Scheme S5. Synthesis of the Tf-TAPPM-COF.

Synthesis of the Tf-TAPPM-COF: The synthesis procedure of the Tf-TAPPM-COF was completely same with that of Tf-TAPB-COF, besides of using TAPPM (35.3 mg, 0.1 mmol) as a substrate. Finally, a brown powder was obtained with a 72% yield.

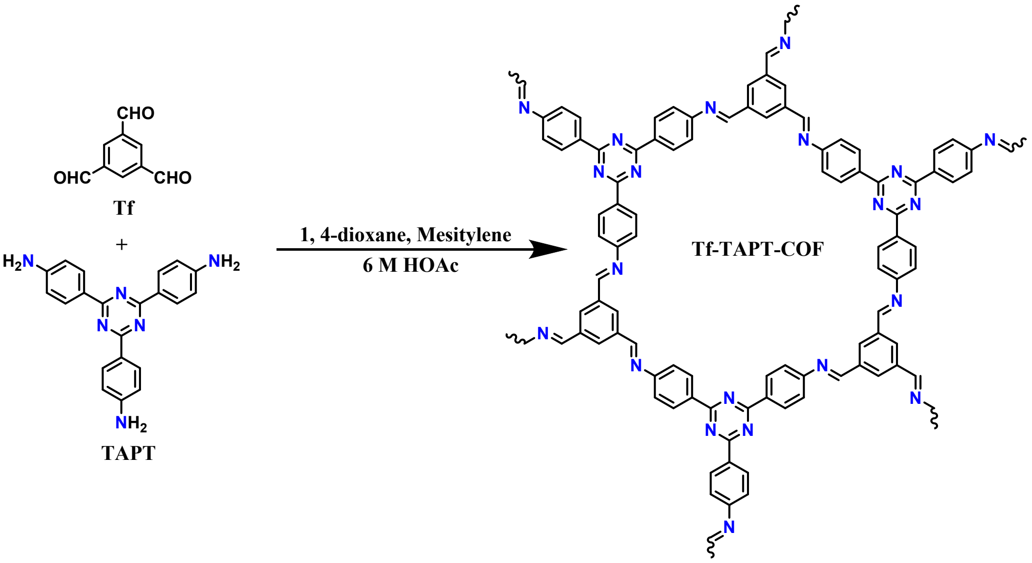

Scheme S6. Synthesis of the Tf-TAPT-COF.

Synthesis of the Tf-TAPT-COF: The Tf-TAPT-COF was synthesized according to a previously published procedure with a slight modification.[7] After adjustment, the synthesis procedure of Tf-TAPT-COF remained consistent with Tf-TAPB-COF, besides of using TAPT (35.4 mg, 0.1 mmol) as a substrate. Finally, a yellow powder was obtained with an 81% yield.

3. Supporting Figures

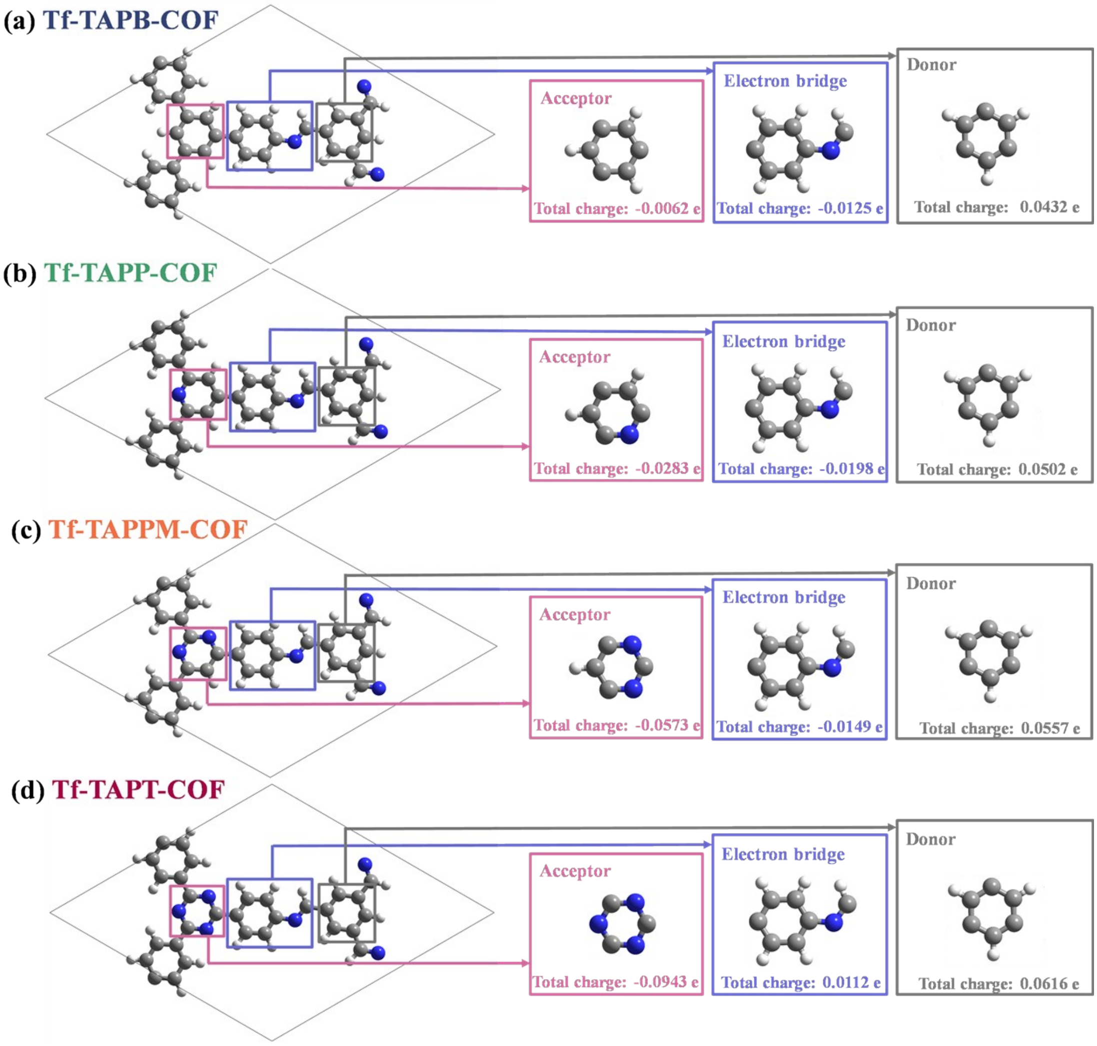

Figure S1. DFT calculation on Hirshfeld charge distribution in (a) Tf-TAPB-COF, (b) Tf-TAPP-COF, (c) Tf-TAPPM-COF,and (d) Tf-TAPT-COF where gray, white, and blue spheres represent C, H, and N atoms, respectively.

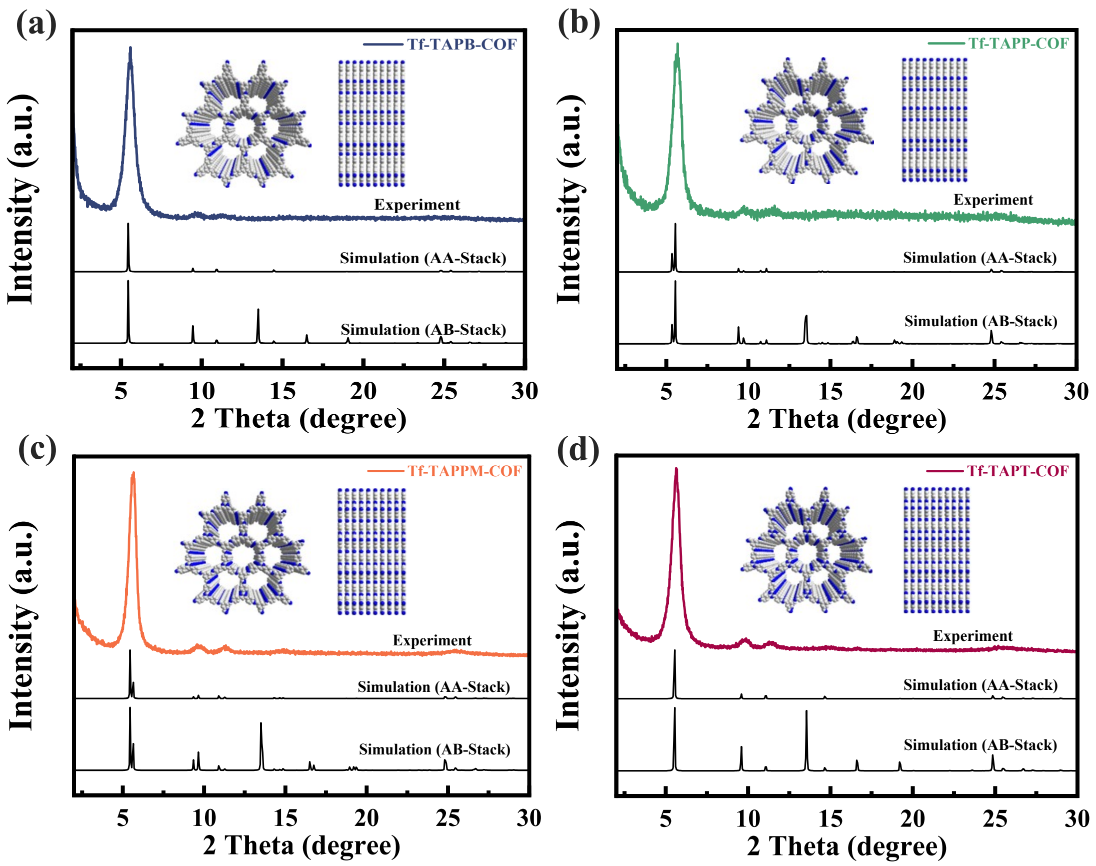

Figure S2. Experimental and simulated powder X-ray diffraction (PXRD) patterns of (a) Tf-TAPB-COF, (b) Tf-TAPP-COF, (c) Tf-TAPPM-COF, and (d) Tf-TAPT-COF.

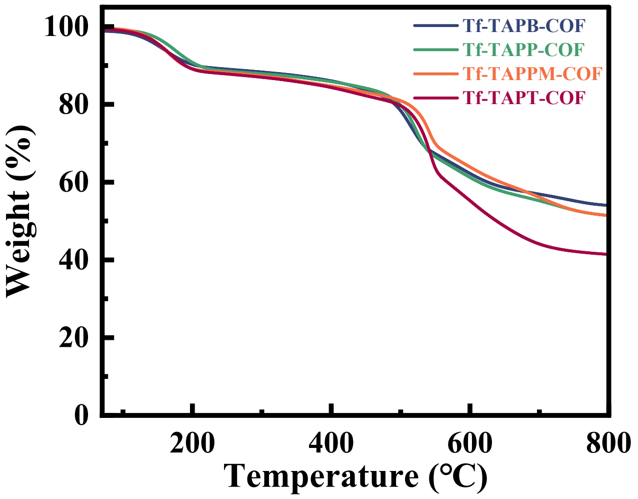

Figure S3. The TGA curves of Tf-TAPB-COF, Tf-TAPP-COF, Tf-TAPPM-COF and Tf-TAPT-COF.

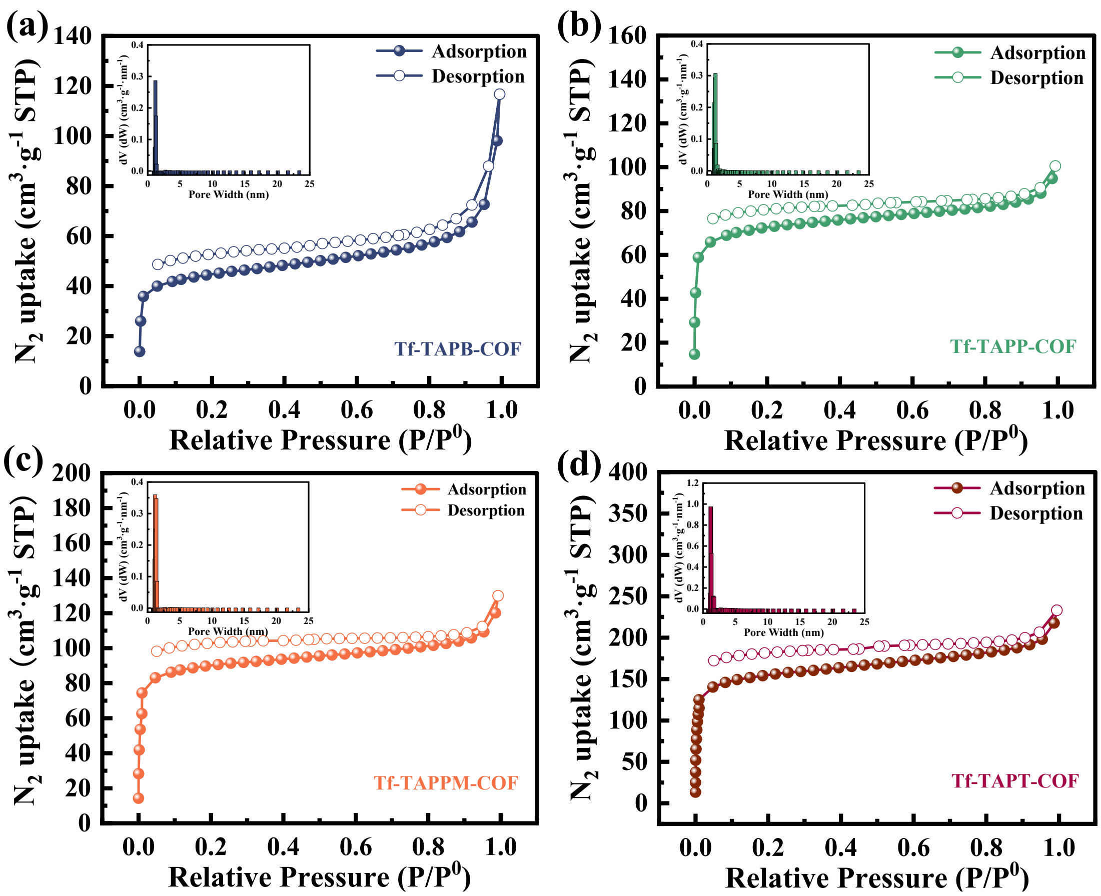

Figure S4. The N_2 adsorption-desorption isotherms and pore size distribution of (a) Tf-TAPB-COF, (b) Tf-TAPP-COF, (c) Tf-TAPPM-COF, and (d) Tf-TAPT-COF.

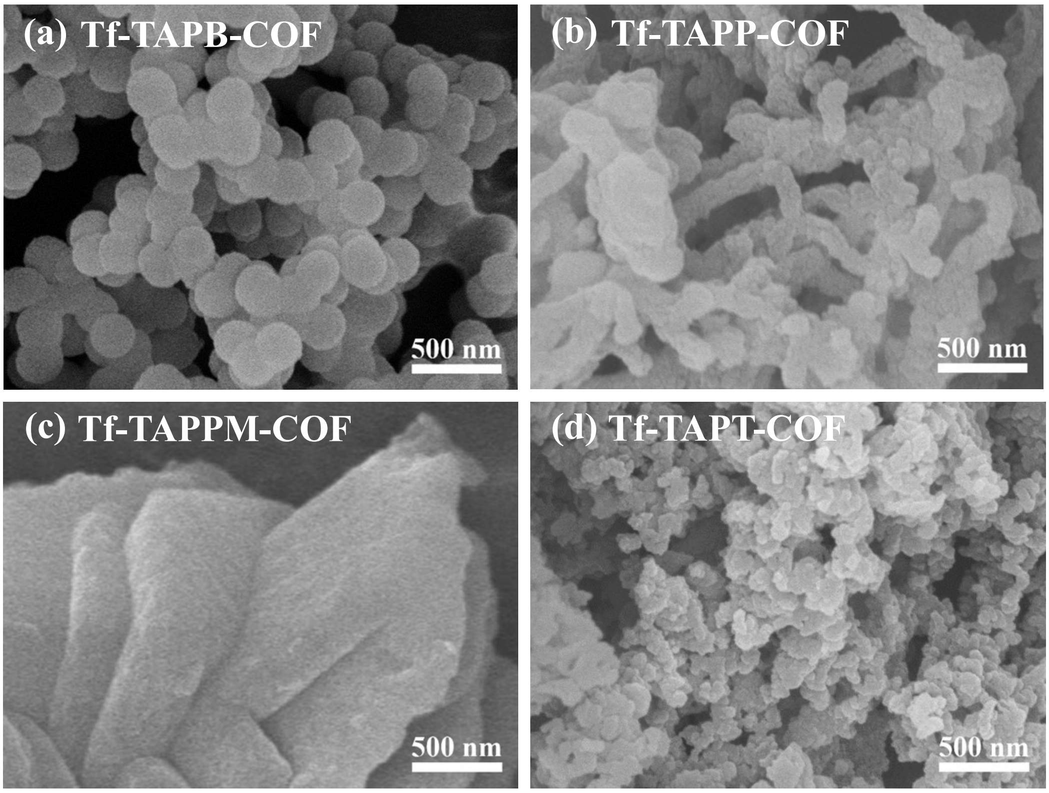

Figure S5. Scanning electron microscopy (SEM) image of (a) Tf-TAPB-COF, (b) Tf-TAPP-COF, (c) Tf-TAPPM-COF, and (d) Tf-TAPT-COF.

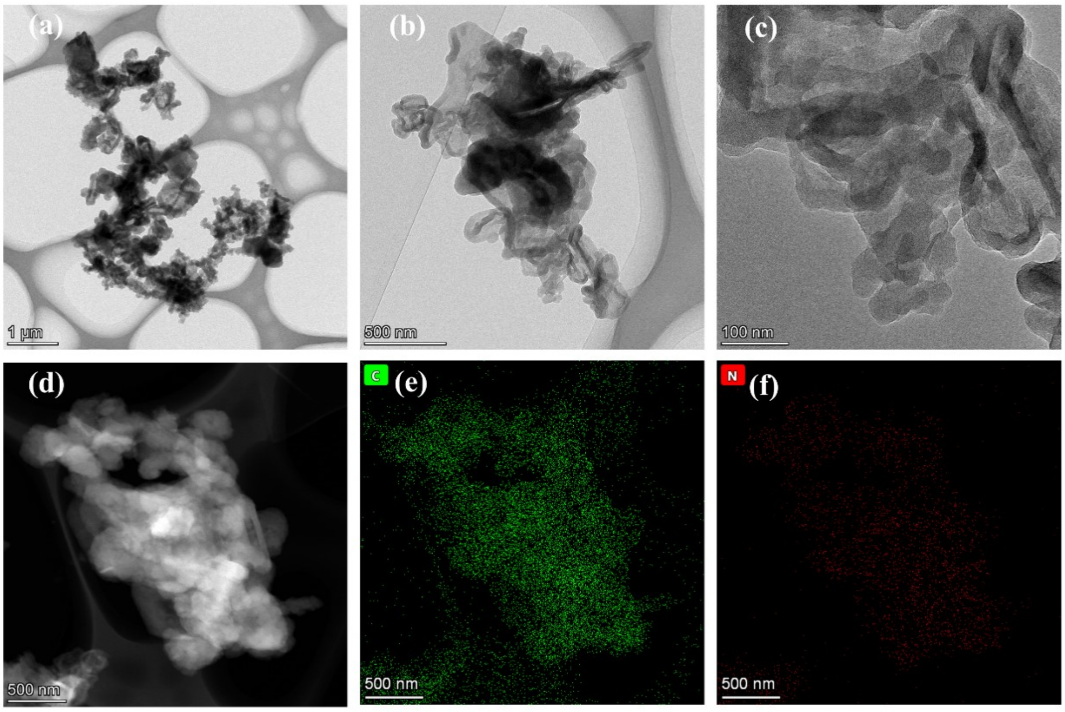

Figure S6. Transmission electron microscopy (TEM) images and EDS element mapping results of Tf-TAPT-COF.

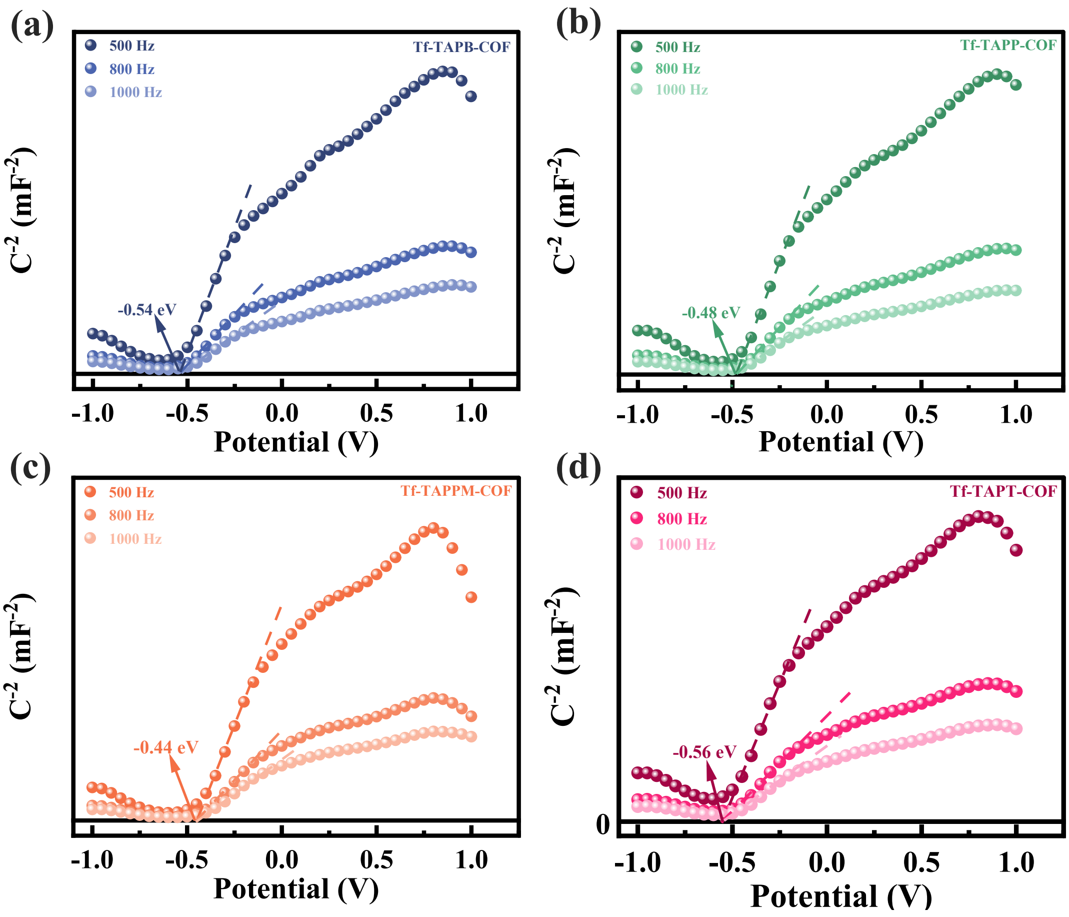

Figure S7. Mott-Schottky plots of (a) Tf-TAPB-COF, (b) Tf-TAPP-COF, (c) Tf-TAPPM-COF, and (d) Tf-TAPT-COF.

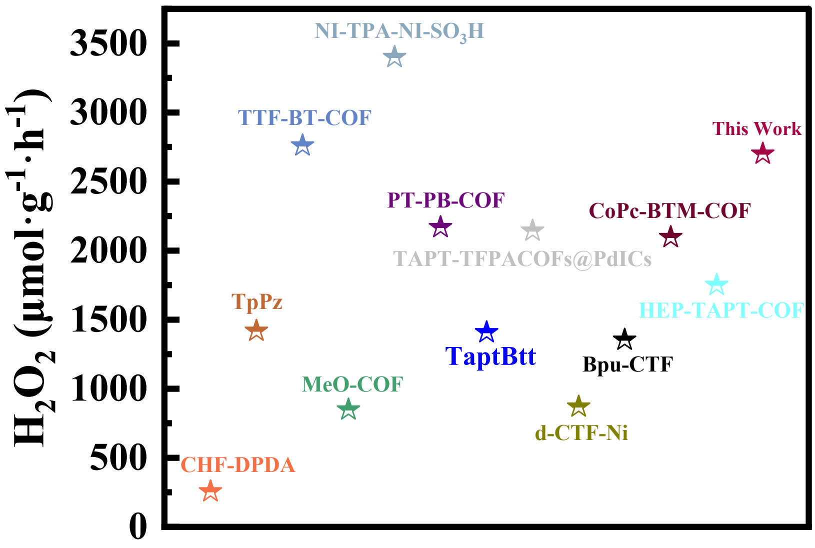

Figure S8. Comparison of the activity of different catalysts for photocatalytic H_2O_2 production.

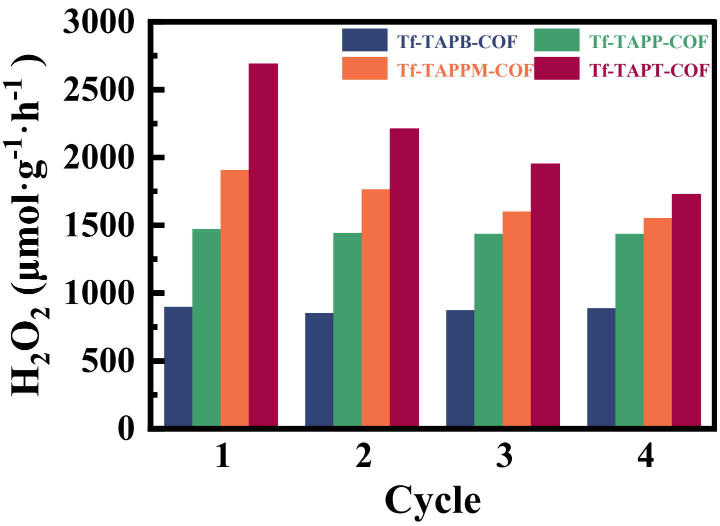

Figure S9. The stability of H_2O_2 evolution for Tf-TAPB-COF, Tf-TAPP-COF, Tf-TAPPM-COF and Tf-TAPT-COF under illumination of a 300 W Xe lamp (λ = 350 - 780 nm).

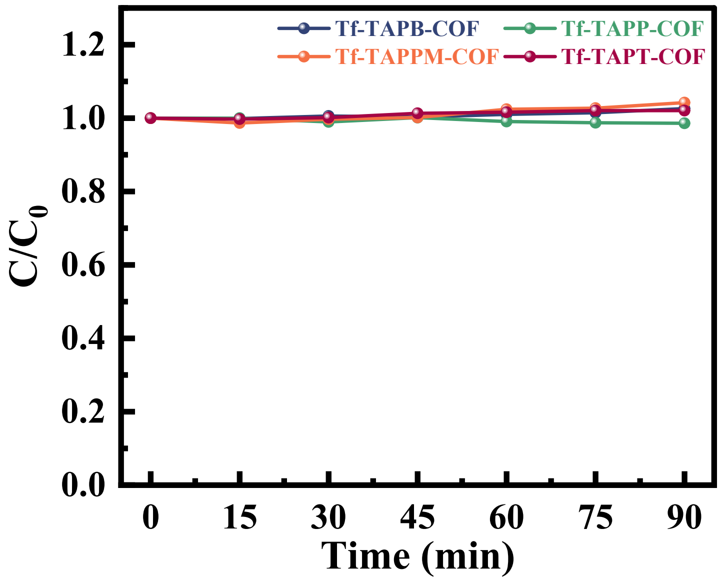

Figure S10. Photocatalytic decomposition of H_2O_2 (C_0 = 1 mM) in pure water under visible light irradiation over Tf-TAPB-COF, Tf-TAPP-COF, Tf-TAPPM-COF and Tf-TAPT-COF.

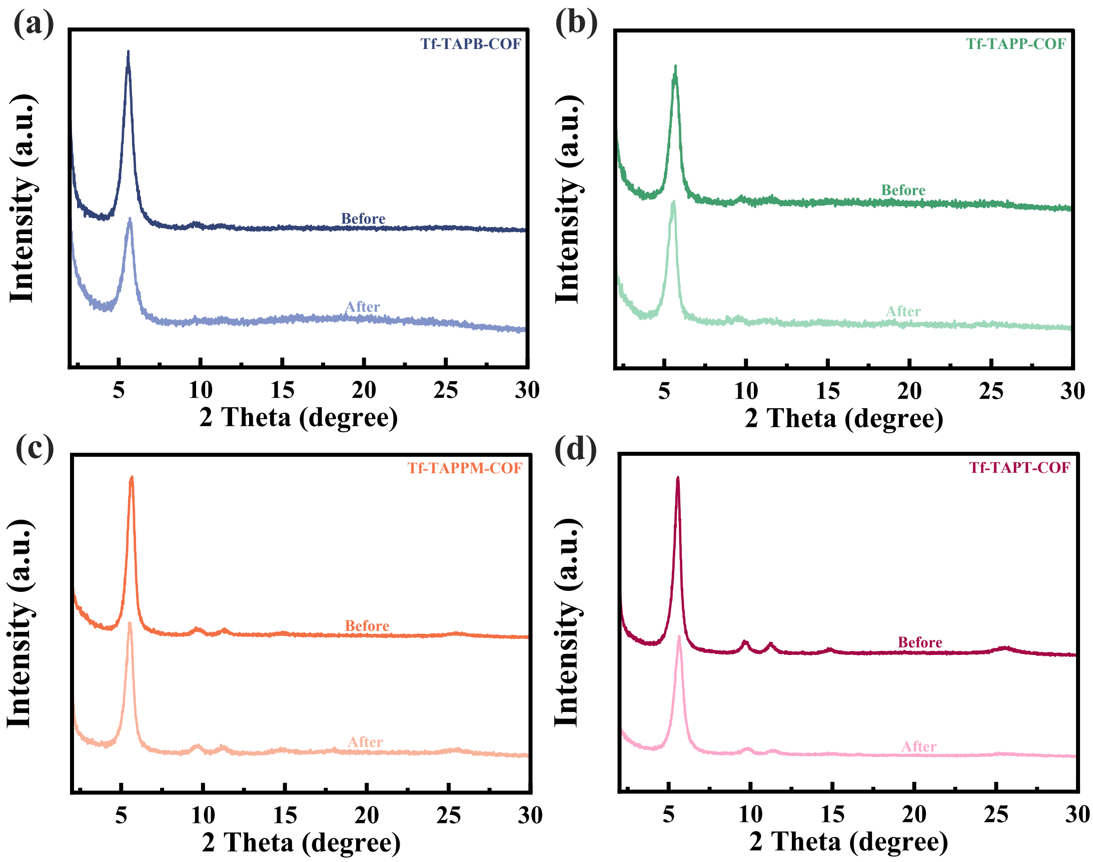

Figure S11. The PXRD patterns before and after photocatalytic reaction of (a) Tf-TAPB-COF, (b) Tf-TAPP-COF, (c) Tf-TAPPM-COF, and (d) Tf-TAPT-COF

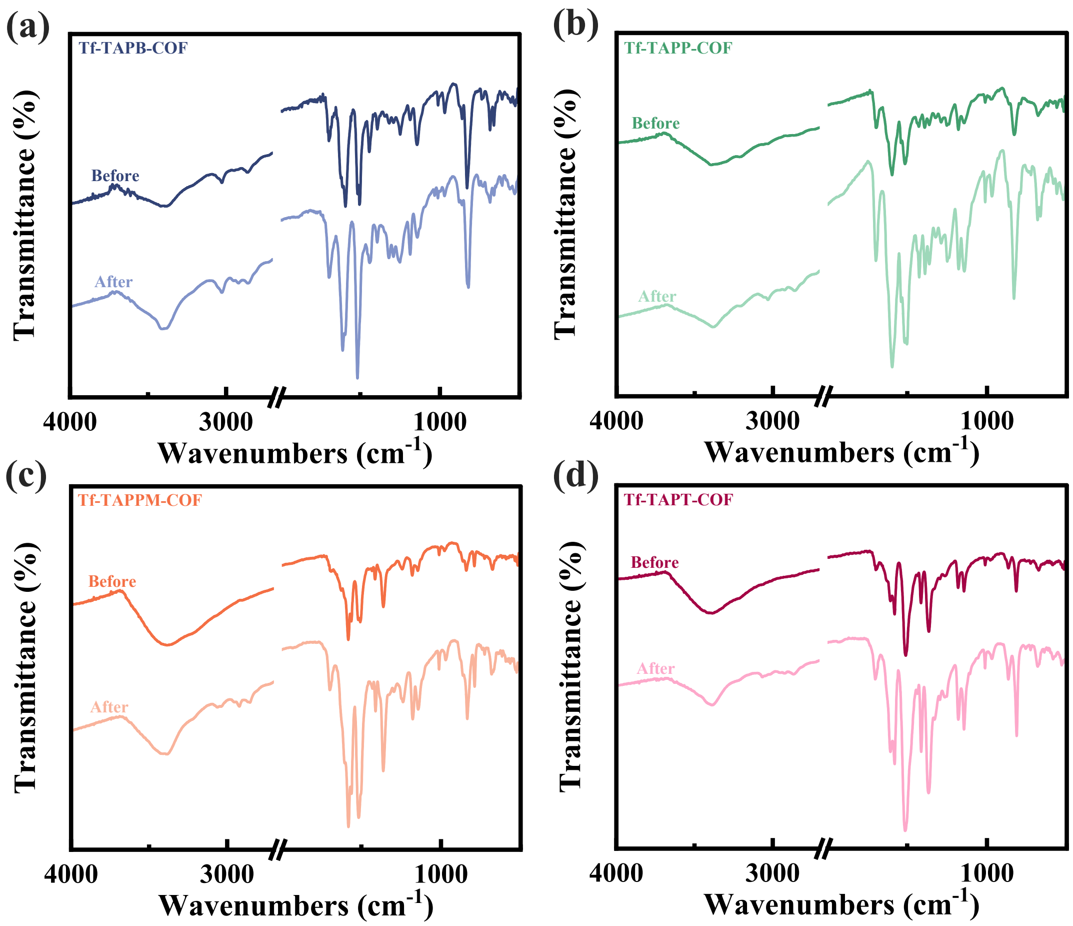

Figure S12. The FT-IR spectra before and after photocatalytic reaction of (a) Tf-TAPB-COF, (b) Tf-TAPP-COF, (c) Tf-TAPPM-COF, and (c) Tf-TAPT-COF.

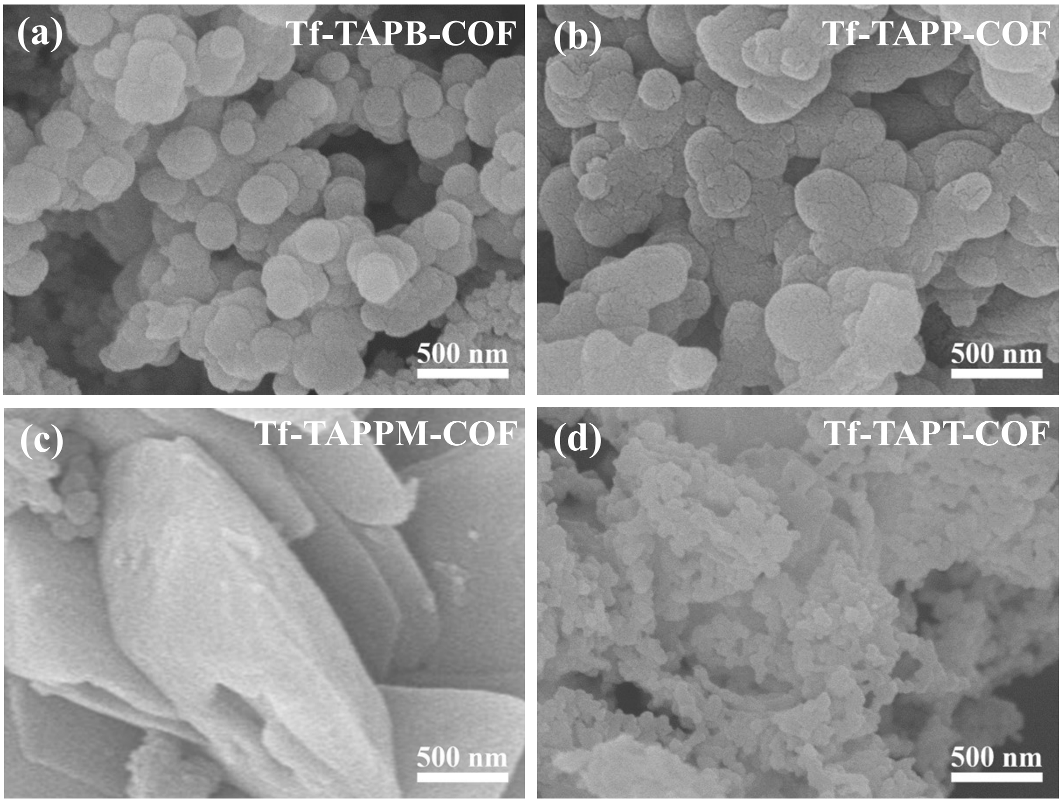

Figure S13. The SEM images of after photocatalytic reaction (a) Tf-TAPB-COF, (b) Tf-TAPP-COF, (c) Tf-TAPPM-COF, and (d) Tf-TAPT-COF.

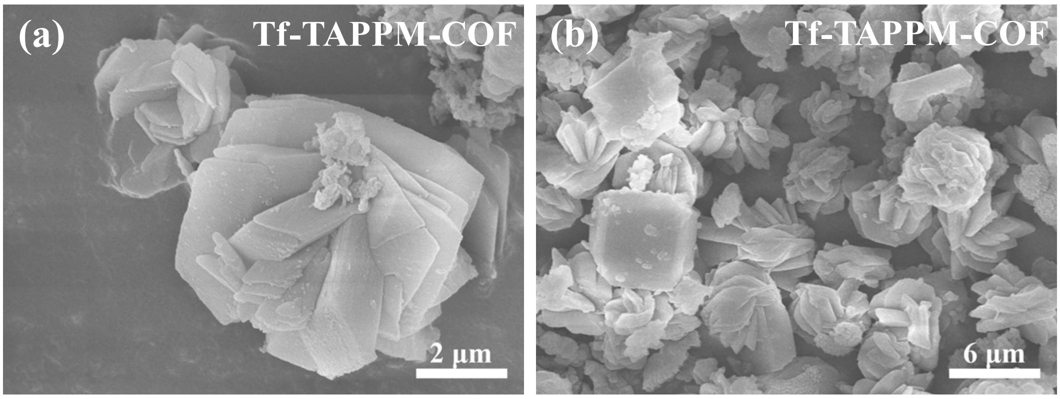

Figure S14. The SEM images of Tf-TAPPM-COF with different size after photocatalytic reaction (a) 2 μm, and (b) 6 μm.

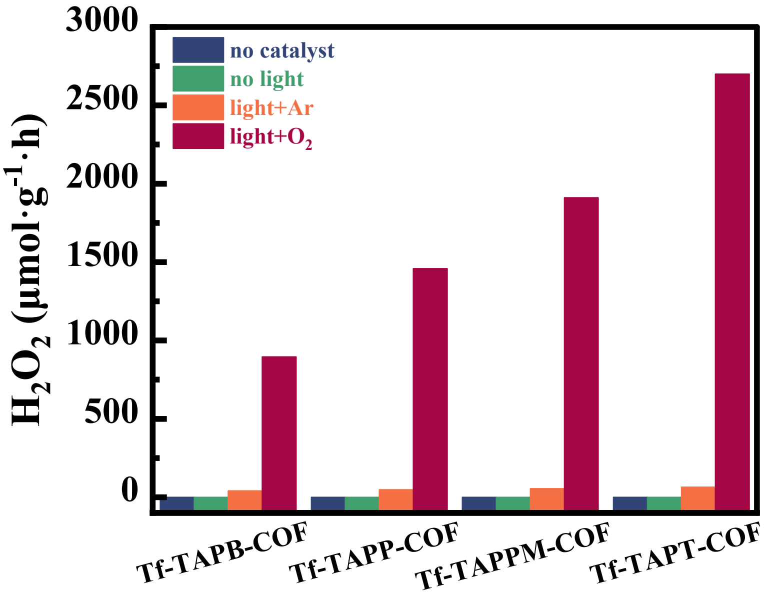

Figure S15. Comparison of activity for photocatalytic H_2O_2 production of Tf-TAPB-COF, Tf-TAPP-COF, Tf-TAPPM-COF, and Tf-TAPT-COF under different conditions.

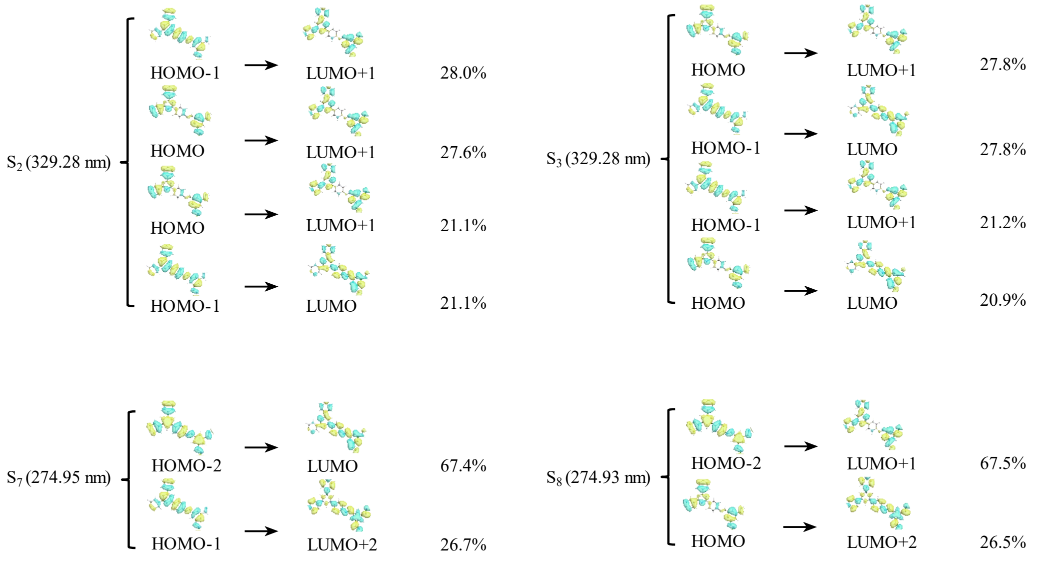

Figure S16. Calculated electronic excitations of Tf-TAPB-COF (TD-DFT calculation at B3LYP, 6-31G level).

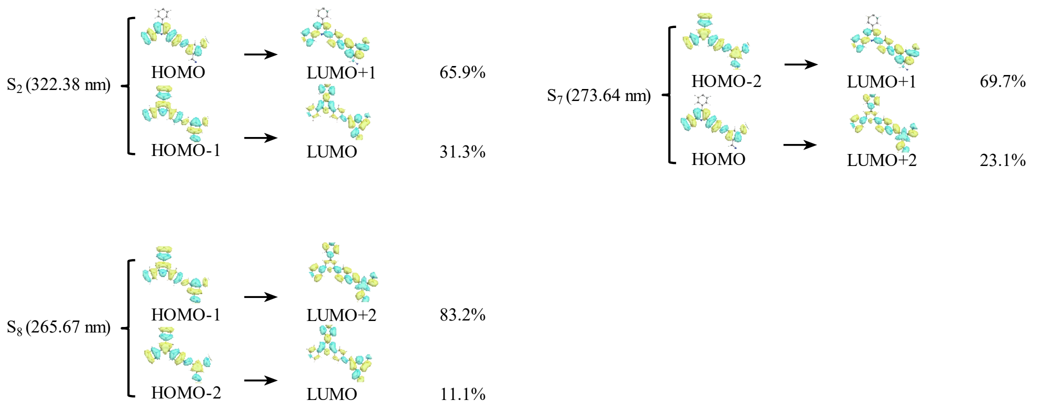

Figure S17. Calculated electronic excitations of Tf-TAPP-COF (TD-DFT calculation at B3LYP, 6-31G level).

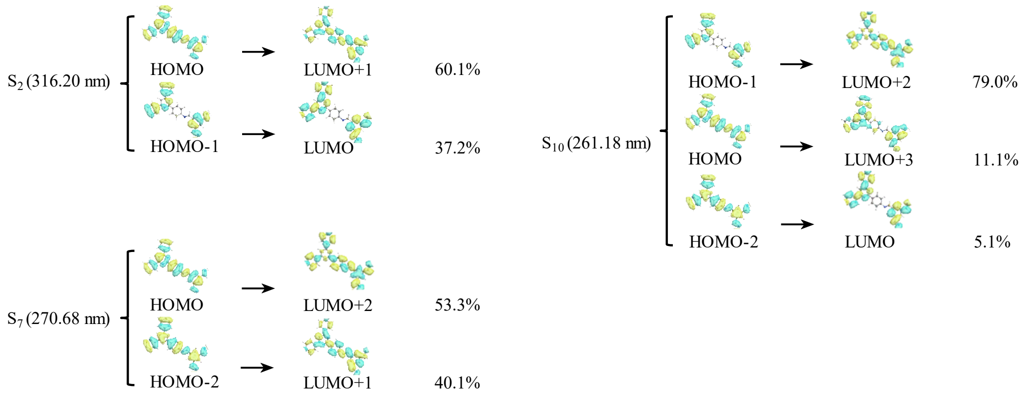

Figure S18. Calculated electronic excitations of Tf-TAPPM-COF (TD-DFT calculation at B3LYP, 6-31G level).

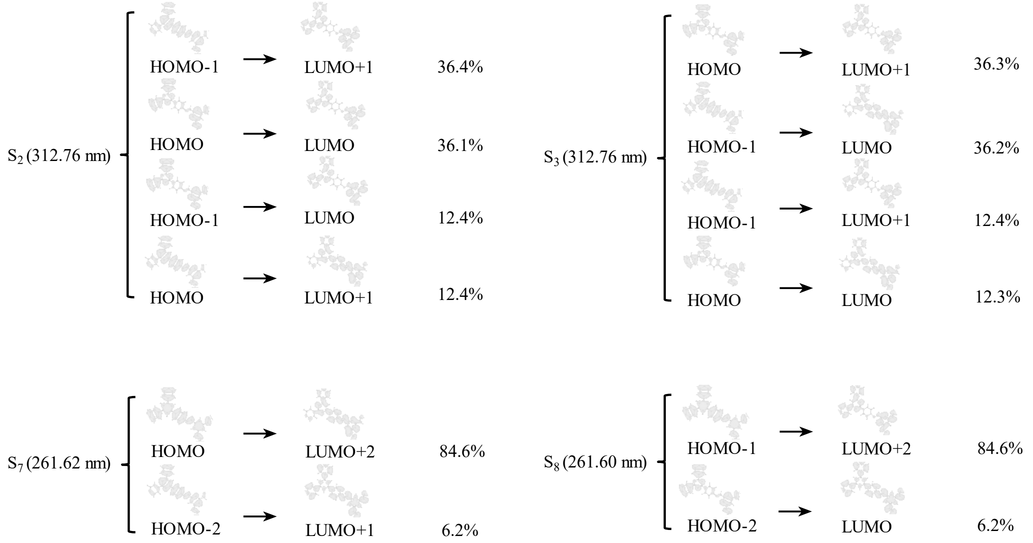

Figure S19. Calculated electronic excitations of Tf-TAPT-COF (TD-DFT calculation at B3LYP, 6-31G level).

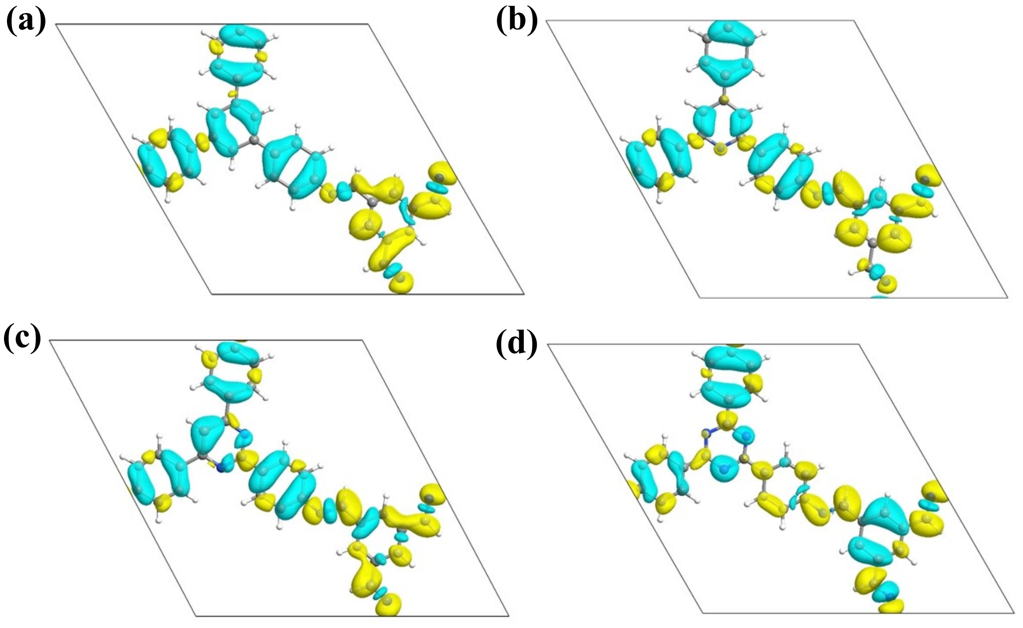

Figure S20. The calculated charge density difference (CDD) of (a) Tf-TAPB-COF, (b) Tf-TAPP-COF, (c) Tf-

TAPPM-COF, and (d) Tf-TAPT-COF, the yellow and blue areas denote charge accumulation and charge loss, respectively.

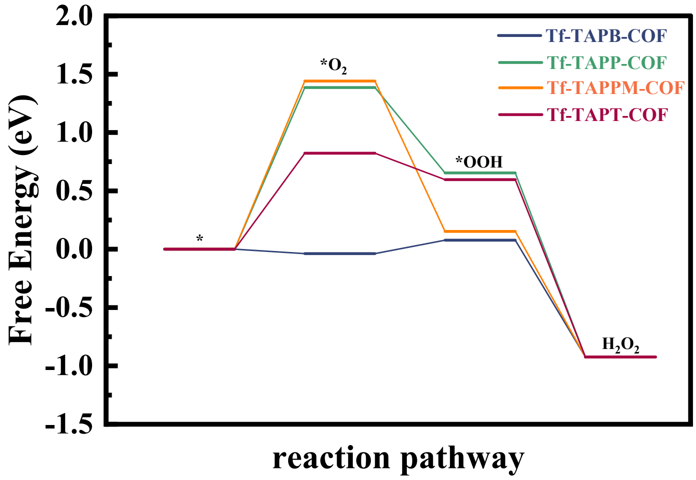

Figure S21. Reduction of oxygen into H_2O_2 on Tf-TAPB-COF, Tf-TAPP-COF, Tf-TAPPM-COF, and Tf-TAPT-COF.

Table S1. Fractional atomic coordinated for unit cell of the Tf-TAPB-COF calculated after performing the Pawley Refinement and Hirshfeld charge distribution.

Tf-TAPB-COF				
**Calculated cell parameters**	a = 18.73355, b = 18.72472, c = 20.00000			
**atoms**	**x**	**y**	**z**	**charge**
N1	11.28524	0.97778	10.00000	-0.11971854
N2	13.63748	6.96859	10.00000	-0.11958225
N3	7.27406	6.00726	10.00000	-0.11951291
C1	-2.60084	6.73304	10.00000	-0.05238441
C2	-1.36582	7.36356	10.00000	-0.04521045
C3	-1.23774	8.76973	10.00000	-0.00031743
C4	-2.43657	9.50947	10.00000	-0.04616165
C5	-3.67817	8.88542	10.00000	-0.04653288
C6	-3.79378	7.48446	10.00000	0.02490623
C7	9.62604	5.52369	10.00000	-0.00819870
C8	9.43720	4.12667	10.00000	-0.02766137
C9	1.27727	8.67283	10.00000	-0.04541995
C10	2.54800	9.26965	10.00000	-0.00096819
C11	8.48705	6.43958	10.00000	0.02554829
C12	6.22885	8.28501	10.00000	-0.05241778
C13	5.06503	9.03860	10.00000	-0.04522666
C14	3.78392	8.44545	10.00000	-0.00028497
C15	3.74291	7.03722	10.00000	-0.04606641
C16	4.90462	6.27475	10.00000	-0.04649927
C17	6.17517	6.87627	10.00000	0.02487414
C18	10.53234	3.25838	10.00000	-0.00820538

C19	11.83671	3.79285	10.00000	-0.02764962
C20	2.61072	10.67207	10.00000	-0.04544262
C21	1.45831	11.47405	10.00000	-0.00098406
C22	10.30628	1.81442	10.00000	0.02552435
C23	0.47455	15.15601	10.00000	-0.05239071
C24	0.40068	13.77138	10.00000	-0.04509931
C25	1.55373	12.95637	10.00000	-0.00030105
C26	2.79464	13.62280	10.00000	-0.04615695
C27	2.87718	15.00984	10.00000	-0.04658317
C28	1.72258	15.81165	10.00000	0.02482589
C29	12.04061	5.17539	10.00000	-0.00821618
C30	10.92561	6.03803	10.00000	-0.02767219
C31	0.21248	10.82731	10.00000	-0.04542164
C32	0.09398	9.42824	10.00000	-0.00092436
C33	13.40399	5.70220	10.00000	0.02558156
H1	-0.45496	15.72590	10.00000	0.05041834
H2	-0.59021	13.31880	10.00000	0.04892837
H3	3.72638	13.05775	10.00000	0.04944044
H4	3.84555	15.51230	10.00000	0.05311690
H5	-2.62710	5.64305	10.00000	0.05044651
H6	-0.47749	6.73333	10.00000	0.04877873
H7	-2.41508	10.59905	10.00000	0.04943701
H8	-4.59834	9.47157	10.00000	0.05317625
H9	7.18591	8.80731	10.00000	0.05039082
H10	5.16599	10.12307	10.00000	0.04877994
H11	2.78878	6.51059	10.00000	0.04955064
H12	4.85867	5.18468	10.00000	0.05320866
H13	1.20880	7.58864	10.00000	0.04433123
H14	3.58385	11.15475	10.00000	0.04425374
H15	-0.69206	11.42898	10.00000	0.04433235
H16	8.75042	7.51336	10.00000	0.04542897
H17	9.24397	1.50819	10.00000	0.04541225
H18	14.20086	4.93599	10.00000	0.04548432
H19	8.41529	3.74260	10.00000	0.05021660
H20	12.68044	3.09993	10.00000	0.05033400
H21	11.10455	7.11499	10.00000	0.05024684

Table S2. Fractional atomic coordinated for unit cell of the Tf-TAPP-COF calculated after performing the Pawley Refinement and Hirshfeld charge distribution.

**Tf-TAPP-COF**				
**Calculated cell parameters**	a = 18.26372, b = 18.82867, c = 20.00000			
**atoms**	**x**	**y**	**z**	**charge**
N1	11.01612	0.97444	10.00000	-0.11936693
N2	13.34185	6.98047	10.00000	-0.11715999
N3	7.00396	6.04782	10.00000	-0.12064781
N4	1.24563	8.90927	10.00000	-0.11620800
C1	-2.41244	6.85036	10.00000	-0.05116584
C2	-1.20945	7.54332	10.00000	-0.04136813
C3	-1.17061	8.95287	10.00000	-0.00655851
C4	-2.39797	9.63998	10.00000	-0.04629771
C5	-3.60324	8.95158	10.00000	-0.04801728
C6	-3.64106	7.54559	10.00000	0.02640139
C7	9.35541	5.51598	10.00000	-0.00749681
C8	9.16728	4.11995	10.00000	-0.02632165
C9	2.44561	9.52032	10.00000	0.04592132
C10	8.22885	6.44570	10.00000	0.02559541
C11	6.07252	8.37011	10.00000	-0.05440744
C12	4.95190	9.18493	10.00000	-0.04553868
C13	3.64781	8.64966	10.00000	-0.00746696
C14	3.51946	7.24965	10.00000	-0.04201669
C15	4.64123	6.42768	10.00000	-0.04539035
C16	5.94142	6.96570	10.00000	0.02656247

C17	10.26613	3.25552	10.00000	-0.00816579
C18	11.57086	3.79006	10.00000	-0.02719812
C19	2.56411	10.91884	10.00000	-0.05304065
C20	1.42292	11.73168	10.00000	0.00868439
C21	10.03895	1.81288	10.00000	0.02876820
C22	0.43401	15.40619	10.00000	-0.05087289
C23	0.36080	14.02154	10.00000	-0.04218368
C24	1.51612	13.21126	10.00000	-0.00139350
C25	2.75819	13.87416	10.00000	-0.04270921
C26	2.83942	15.26053	10.00000	-0.04487496
C27	1.68309	16.05993	10.00000	0.02887726
C28	11.77297	5.17245	10.00000	-0.00742326
C29	10.65508	6.03030	10.00000	-0.02610666
C30	0.18951	11.06763	10.00000	-0.05314919
C31	0.13188	9.66567	10.00000	0.04586048
C32	13.13372	5.71049	10.00000	0.02604034
H1	-0.49496	15.97669	10.00000	0.05180712
H2	-0.62859	13.56483	10.00000	0.05047052
H3	3.68835	13.30676	10.00000	0.05116839
H4	3.80631	15.76538	10.00000	0.05476729
H5	-2.39067	5.75974	10.00000	0.05074154
H6	-0.26227	7.00495	10.00000	0.04732736
H7	-2.42926	10.72911	10.00000	0.04906660
H8	-4.55517	9.48422	10.00000	0.05223393
H9	7.05547	8.84113	10.00000	0.04944540
H10	5.10789	10.26333	10.00000	0.04825591
H11	2.51784	6.82144	10.00000	0.04785186
H12	4.53954	5.34119	10.00000	0.05316195
H13	3.55043	11.37179	10.00000	0.04686441
H14	-0.73398	11.63793	10.00000	0.04681600
H15	8.51884	7.51222	10.00000	0.04475386
H16	8.97589	1.50979	10.00000	0.04626910
H17	13.94206	4.95595	10.00000	0.04663572
H18	8.14734	3.73039	10.00000	0.05180754
H19	12.41693	3.10045	10.00000	0.05093363
H20	10.82835	7.10832	10.00000	0.05018200

Table S3. Fractional atomic coordinated for unit cell of the Tf-TAPPM-COF calculated after performing the Pawley Refinement and Hirshfeld charge distribution.

Tf-TAPPM-COF				
**Calculated cell parameters**	a = 18.36635, b = 18.36388, c = 20.00000			
**atoms**	**x**	**y**	**z**	**charge**
N1	11.20293	0.91079	10.00000	-0.12038775
N2	13.67184	6.88748	10.00000	-0.11667013
N3	7.32013	6.04195	10.00000	-0.11790324
N4	1.49875	8.73242	10.00000	-0.12323863
N5	2.85945	10.70613	10.00000	-0.12340727
C1	-2.18827	6.71469	10.00000	-0.04955834
C2	-0.97451	7.38797	10.00000	-0.03796022
C3	-0.91942	8.79665	10.00000	-0.00769413
C4	-2.13346	9.50611	10.00000	-0.04279367
C5	-3.34846	8.83660	10.00000	-0.04630077
C6	-3.40525	7.43099	10.00000	0.03027601
C7	9.66142	5.50514	10.00000	-0.00681374
C8	9.44910	4.11228	10.00000	-0.02485470
C9	2.68305	9.37208	10.00000	0.09207931
C10	8.54189	6.44698	10.00000	0.02640277
C11	6.32739	8.34413	10.00000	-0.05337683
C12	5.18008	9.12320	10.00000	-0.04146732
C13	3.90233	8.53049	10.00000	-0.01254858
C14	3.80890	7.12896	10.00000	-0.04178772
C15	4.95821	6.34799	10.00000	-0.04690122
C16	6.23773	6.93489	10.00000	0.02819606

C17	10.52949	3.22485	10.00000	-0.00754907
C18	11.84422	3.73232	10.00000	-0.02603723
C19	1.75387	11.47588	10.00000	0.05456174
C20	10.26177	1.79012	10.00000	0.02888509
C21	1.01123	15.19844	10.00000	-0.05285147
C22	0.83806	13.82438	10.00000	-0.04244530
C23	1.93874	12.94414	10.00000	-0.00854647
C24	3.22898	13.50271	10.00000	-0.03811772
C25	3.40631	14.88198	10.00000	-0.04375738
C26	2.30570	15.75952	10.00000	0.03052135
C27	12.07090	5.11052	10.00000	-0.00748857
C28	10.97057	5.99303	10.00000	-0.02582409
C29	0.48251	10.88876	10.00000	-0.06134807
C30	0.38691	9.49169	10.00000	0.05447664
C31	13.44156	5.62128	10.00000	0.02946431
H1	0.12617	15.83437	10.00000	0.05078550
H2	-0.18003	13.43616	10.00000	0.04999176
H3	4.08606	12.82947	10.00000	0.05011513
H4	4.40553	15.31932	10.00000	0.05467547
H5	-2.18630	5.62372	10.00000	0.05215212
H6	-0.03498	6.83579	10.00000	0.04954771
H7	-2.14238	10.59569	10.00000	0.05079958
H8	-4.29276	9.38158	10.00000	0.05385143
H9	7.29809	8.84046	10.00000	0.04965541
H10	5.24826	10.21104	10.00000	0.04709514
H11	2.82050	6.67039	10.00000	0.04775737
H12	4.89850	5.25906	10.00000	0.05231516
H13	-0.41211	11.50352	10.00000	0.04954161
H14	8.83117	7.51372	10.00000	0.04614445
H15	9.18802	1.52790	10.00000	0.04566325
H16	14.23567	4.85227	10.00000	0.04775794
H17	8.42103	3.74406	10.00000	0.05153098
H18	12.67661	3.02592	10.00000	0.05227085
H19	11.16826	7.06694	10.00000	0.05050917

Table S4. Fractional atomic coordinated for unit cell of Tf-TAPT-COF calculated after performing the Pawley Refinement and Hirshfeld charge distribution.

Tf-TAPT-COF				
**Calculated cell parameters**	a = 18.47649, b = 18.46633, c = 20.00000			
**atoms**	**x**	**y**	**z**	**charge**
N1	11.13080	0.91406	10.00000	-0.11741527
N2	13.49612	6.89772	10.00000	-0.11745275
N3	7.13128	5.94952	10.00000	-0.11746958
N4	1.25802	8.54673	10.00000	-0.13131374
N5	2.58181	10.52682	10.00000	-0.13112250
N6	0.20494	10.68346	10.00000	-0.13132376
C1	-2.48824	6.65346	10.00000	-0.05163589
C2	-1.25183	7.27968	10.00000	-0.03803405
C3	-1.15457	8.68431	10.00000	-0.01340987
C4	-2.33458	9.44698	10.00000	-0.03771612
C5	-3.57421	8.81989	10.00000	-0.04493063
C6	-3.67727	7.41517	10.00000	0.03236803
C7	9.48161	5.46157	10.00000	-0.00681945
C8	9.28930	4.06504	10.00000	-0.02457612
C9	2.44075	9.18826	10.00000	0.09980978
C10	8.34434	6.38090	10.00000	0.03006469
C11	6.09472	8.22898	10.00000	-0.05164081
C12	4.93414	8.98656	10.00000	-0.03803931
C13	3.66906	8.36843	10.00000	-0.01340493
C14	3.59957	6.96501	10.00000	-0.03776041

C15	4.76254	6.20486	10.00000	-0.04496102
C16	6.03054	6.81822	10.00000	0.03232429
C17	10.38251	3.19498	10.00000	-0.00683611
C18	11.68809	3.72683	10.00000	-0.02447689
C19	1.43476	11.23044	10.00000	0.09982633
C20	10.15271	1.75122	10.00000	0.02994236
C21	0.44323	14.87648	10.00000	-0.05165720
C22	0.36472	13.49266	10.00000	-0.03803760
C23	1.53125	12.70403	10.00000	-0.01343854
C24	2.78260	13.34266	10.00000	-0.03777520
C25	2.86212	14.72951	10.00000	-0.04491936
C26	1.69864	15.52327	10.00000	0.03235437
C27	11.89566	5.10870	10.00000	-0.00677748
C28	10.78215	5.97333	10.00000	-0.02449368
C29	0.16932	9.33827	10.00000	0.09980767
C30	13.26117	5.63193	10.00000	0.03001730
H1	-0.48103	15.45497	10.00000	0.05119864
H2	-0.60256	12.99100	10.00000	0.04916106
H3	3.68371	12.73058	10.00000	0.05004332
H4	3.82652	15.23956	10.00000	0.05430328
H5	-2.52550	5.56361	10.00000	0.05122384
H6	-0.33270	6.69450	10.00000	0.04914389
H7	-2.25728	10.53364	10.00000	0.05013040
H8	-4.49935	9.39827	10.00000	0.05424998
H9	7.05666	8.74225	10.00000	0.05122005
H10	4.98145	10.07512	10.00000	0.04917099
H11	2.62022	6.48797	10.00000	0.05005298
H12	4.72375	5.11450	10.00000	0.05426063
H13	8.61001	7.45329	10.00000	0.04733994
H14	9.09055	1.44765	10.00000	0.04724255
H15	14.05556	4.86426	10.00000	0.04735402
H16	8.26604	3.68445	10.00000	0.05177624
H17	12.52918	3.03078	10.00000	0.05188648
H18	10.96385	7.04986	10.00000	0.05187064

Table S5. Comparison of photocatalytic H_2O_2 production in pure water by recently reported photocatalysts.

Sample	Time (hour)	Dosage (mg)	H_2O_2 yield (μmol g^{-1} h^{-1})	Reaction conditions	Ref.
CHF-DPDA	6	375	256	Solution volume: 75 mL<br>Xe lamp (λ≥420 nm): 100 mW·cm^{-2}<br>O_2 saturated pure water<br>Temperature: --	8
TpPz	1	3	1418	Solution volume: 18 mL<br>Xe lamp (λ>420 nm): --<br>O_2 saturated pure water<br>Temperature: 293 K	9
TTF-BT-COF	1	5	2760	Solution volume: 10 mL<br>Xe lamp (λ>420 nm): 20.3 mW·cm^{-2}<br>O_2 saturated pure water<br>Temperature: 298 K	10
MeO-COF	1	--	847.9	Solution volume: --<br>Xe lamp (λ>420 nm): --<br>O_2 saturated pure water<br>Temperature: --	11
NI-TPA-NI-SO_3H	1	5	3400	Solution volume: 20 mL<br>Xe lamp (λ>420 nm): 600 mW·cm^{-2}<br>Air pure water<br>Temperature: 298 K	12
PT-PB-COF	1	5	2169	Solution volume: 20 mL<br>Xe lamp (λ>420 nm): 83 mW·cm^{-2}<br>O_2 saturated pure water	13

				Temperature: 298 K	
TaptBtt	7	50	1407	Solution volume: 60 mL<br>Xe lamp (λ≥420 nm): --<br>O_2 saturated pure water<br>Temperature: --	14
TAPT-TFPA COFs@PdICs	3	10	2143	Solution volume: 20 mL<br>Xe lamp (λ>420 nm): --<br>O_2 saturated 10% ethanol water solution<br>Temperature: 298 K	15
d-CTF-Ni	3	20	869.1	Solution volume: 50 mL<br>Xe lamp (λ>420 nm): 100 $mW \cdot cm^{-2}$<br>O_2 saturated pure water<br>Temperature: 298 K	16
Bpu-CTF	1	10	1353	Solution volume: 50 mL<br>Xe lamp (λ>420 nm): 100 $mW \cdot cm^{-2}$<br>O_2 saturated pure water<br>Temperature: 298 K	17
CoPc-BTM-COF	0.67	5	2096	Solution volume: 50 mL<br>Xe lamp (λ>400 nm): 19.2 $mW \cdot cm^{-2}$<br>O_2 saturated 10% ethanol water solution<br>Temperature: 298 K	18
HEP-TAPT-COF	8	50	1750	Solution volume: 100 mL<br>Xe lamp (λ>420 nm): 100 $mW \cdot cm^{-2}$<br>O_2 saturated pure water<br>Temperature: 298 K	19
Tf-TAPT-COF	1.5	10	2700	Solution volume: 50 mL<br>Xe lamp (λ≥420 nm): 63 $mW \cdot cm^{-2}$<br>O_2 saturated pure water<br>Temperature: 298 K	This work

Table S6. Calculated molecular orbital transition (MOT), contribution of transition, excitation energy (E), wavelength (λ) and oscillator strength (f) at different excited states for Tf-TAPB-COF, Tf-TAPP-COF, Tf-TAPPM-COF, and Tf-TAPT-COF.

Model	Excitation	MOT	MO contribution of transition (%)	E (eV)	λ (nm)	f
Tf-TAPB-COF	S0 → S2	HOMO-1 → LUMO+1	28.00	329.28	3.7653	0.64654
		HOMO → LUMO	27.60			
		HOMO → LUMO+1	21.10			
		HOMO-1 → LUMO	21.10			
	S0 → S3	HOMO → LUMO+1	27.80	329.28	3.7653	0.64790
		HOMO-1 → LUMO	27.80			
		HOMO-1 → LUMO+1	21.20			
		HOMO → LUMO	20.90			
	S0 → S5	HOMO → LUMO+2	70.80	295.31	4.1985	0.07070
		HOMO-2 → LUMO+1	28.60			
	S0 → S6	HOMO-1 → LUMO+2	70.60	295.28	4.1988	0.06936
		HOMO-2 → LUMO	28.80			

	S0 → S7	HOMO-2 → LUMO	67.40	274.95	4.5094	0.95462
		HOMO-1 → LUMO+2	26.70			
	S0 → S8	HOMO-2 → LUMO+1	67.50	274.93	4.5097	0.95535
		HOMO → LUMO+2	26.50			
Tf-TAPP-COF	S0 → S1	HOMO → LUMO	95.70	351.23	3.5300	0.22622
	S0 → S2	HOMO → LUMO+1	65.90	322.38	3.8459	0.67919
		HOMO-1 → LUMO	31.30			
	S0 → S3	HOMO-1 → LUMO+1	94.10	307.95	4.0261	0.35518
	S0 → S4	HOMO-1 → LUMO+2	79.50	294.87	4.2048	0.12226
		HOMO-2 → LUMO+1	11.30			
	S0 → S5	HOMO-2 → LUMO	79.50	292.55	4.2380	0.15353
		HOMO-1 → LUMO+2	11.30			
	S0 → S6	HOMO-1 → LUMO	48.20	288.18	4.3024	0.10983
		HOMO-2 → LUMO+2	21.00			
		HOMO → LUMO+1	18.00			
		HOMO-2 → LUMO	6.80			
	S0 → S7	HOMO-2 → LUMO+1	69.70	273.64	4.5309	0.92501
		HOMO → LUMO+2	23.10			
	S0 → S8	HOMO-1 → LUMO+2	83.20	265.67	4.6668	0.89080
		HOMO-2 → LUMO	11.10			
	S0 → S10	HOMO-3 → LUMO	65.60	255.83	4.8463	0.01251
		HOMO-4 → LUMO	19.70			

Tf-TAPPM-COF	S0 → S1	HOMO → LUMO	95.20	343.66	3.6078	0.26241
	S0 → S2	HOMO → LUMO+1	60.10	316.20	3.9211	0.58545
		HOMO-1 → LUMO	37.20			
	S0 → S3	HOMO-1 → LUMO+1	93.20	304.54	4.0713	0.42442
	S0 → S4	HOMO-2 → LUMO	91.00	294.75	4.2064	0.39447
		HOMO-1 → LUMO+2	6.50			
	S0 → S6	HOMO-1 → LUMO	43.50	284.16	4.3632	0.01066
		HOMO-2 → LUMO+2	26.20			
		HOMO → LUMO+1	24.00			
	S0 → S7	HOMO → LUMO+2	53.30	270.68	4.5804	1.08029
		HOMO-2 → LUMO+1	40.10			
	S0 → S8	HOMO-3 → LUMO	99.40	265.21	4.6749	0.00498
	S0 → S9	HOMO-4 → LUMO	97.80	264.91	4.6802	0.00493
	S0 → S10	HOMO-1 → LUMO+2	79.00	261.18	4.7470	0.71512
		HOMO → LUMO+3	11.10			
		HOMO-2 → LUMO	5.10			
Tf-TAPT-COF	S0 → S2	HOMO-1 → LUMO+1	36.40	312.76	3.9642	0.64422
		HOMO → LUMO	36.10			
		HOMO-1 → LUMO	12.40			
		HOMO → LUMO+1	12.40			
	S0 → S3	HOMO → LUMO+1	36.30	312.76	3.9642	0.64480
		HOMO-1 → LUMO	36.20			
		HOMO-1 →	12.40			

		LUMO+1				
		HOMO → LUMO	12.30			
	S0 → S4	HOMO-2 → LUMO	85.30	293.03	4.2312	0.30045
		HOMO-1 → LUMO+2	6.50			
	S0 → S5	HOMO-2 → LUMO+1	85.20	292.99	4.2312	0.30102
		HOMO → LUMO+2	6.50			
	S0 → S7	HOMO → LUMO+2	84.60	261.62	4.7392	0.82083
		HOMO-2 → LUMO+1	6.20			
	S0 → S8	HOMO-1 → LUMO+2	84.60	261.60	4.7395	0.8189
		HOMO-2 → LUMO	6.20			
	S0 → S9	HOMO-3 → LUMO	28.30	257.63	4.8124	0.00419
		HOMO-5 → LUMO	25.50			
		HOMO-4 → LUMO+1	25.10			
		HOMO-5 → LUMO+1	14.40			
	S0 → S10	HOMO-3 → LUMO+1	31.50	257.62	4.8127	0.00447
		HOMO-5 → LUMO+1	26.90			
		HOMO-5 → LUMO	25.10			
		HOMO-4 → LUMO	12.40			

Table S7. Quantitative analysis of charge carrier dynamics in four COFs: the electro-hole overlap (S_m, defined as the minimum of holes and electrons), oscillator strength (f), the molecular polarity index (MPI), Polar surface area, and Nonpolar surface area.

Model	Excitation	S_m	f	MPI (kcal mol^{-1})	Polar surface area	Nonpolar surface area
Tf-TAPB-COF	S0 → S2	0.47484	0.64654	9.37	44.23%	55.77%
	S0 → S3	0.47467	0.64790			
	S0 → S7	0.48801	0.95462			
	S0 → S8	0.48747	0.95535			
Tf-TAPP-COF	S0 → S2	0.52127	0.67919	9.32	40.69%	59.31%
	S0 → S7	0.49311	0.92501			
	S0 → S8	0.48377	0.89080			
Tf-TAPPM-COF	S0 → S2	0.58569	0.58545	8.94	38.46%	61.54%
	S0 → S7	0.53768	1.08029			
	S0 → S10	0.4989	0.71512			
Tf-TAPT-COF	S0 → S2	0.60222	0.64422	8.23	34.68%	65.32%
	S0 → S3	0.60233	0.64480			
	S0 → S7	0.47101	0.82083			
	S0 → S8	0.45916	0.81890			

References

1 N. Lu, N. Liu, Y. Hui, K. Shang, N. Jiang, J. Li and Y. Wu, *Chemosphere*, 2020, **241**, 124927.

2 T. Lu, *J. Chem. Phys.*, 2024, **161**, 82503.

3 T. Lu and F. Chen, *J. Comput. Chem.*, 2012, **33**, 580–592.

4 W. Chen, W. Yan, S. Wu, Z. Xu, K. W. K. Yeung and C. Yi, *Macromol. Chem. Phys.*, 2010, **211**, 1803–1813.

5 D. Chakraborty, S. Nandi, D. Mullangi, S. Haldar, C. P. Vinod and R. Vaidhyanathan, *ACS Appl. Mater. Interfaces*, 2019, **11**, 15670–15679.

6 G. Zhao, Y. Sun, Y. Yang, C. Zhang, Q. An and H. Guo, *EcoMat*, 2022, **4**, e12221.

7 J. Á. Martín-Illán, D. Rodríguez-San-Miguel, O. Castillo, G. Beobide, J. Perez-Carvajal, I. Imaz, D. Maspoch and F. Zamora, *Angew. Chem. Int. Ed.*, 2021, **60**, 13969–13977.

8 H. Cheng, H. Lv, J. Cheng, L. Wang, X. Wu and H. Xu, *Adv. Mater.*, 2022, **34**, 2107480.

9 Q. Liao, Q. Sun, H. Xu, Y. Wang, Y. Xu, Z. Li, J. Hu, D. Wang, H. Li and K. Xi, *Angew. Chem. Int. Ed.*, 2023, **62**, e202310556.

10 J. Chang, Q. Li, J. Shi, M. Zhang, L. Zhang, S. Li, Y. Chen, S. Li and Y. Lan, *Angew. Chem. Int. Ed.*, 2023, **62**, e202218868.

11 C. Li, H. Xie, S. Zhou, H. Hu, G. Chen, Z. Wei, J. Jiang, J. Qin, Z. Zhang and Y. Kong, *Mater. Res. Bull.*, 2024, **173**, 112697.

12 X. Luo, S. Zhou, S. Zhou, X. Zhou, J. Huang, Y. Liu, D. Wang, G. Liu and P. Gu, *Adv. Funct. Mater.*, 2024, 2415244.

13 W. Wang, R. Zhang, H. Chu, Z. Zhan, Q. Huang, Z. Li, X. Wang, F. Bai and W. Zhou, *Small*, 2024, 2406527.

14 C. Qin, X. Wu, L. Tang, X. Chen, M. Li, Y. Mou, B. Su, S. Wang, C. Feng, J. Liu, X. Yuan, Y. Zhao and H. Wang, *Nat. Commun.*, 2023, **14**, 5238.

15 Y. Liu, L. Li, H. Tan, N. Ye, Y. Gu, S. Zhao, S. Zhang, M. Luo and S. Guo, *J. Am. Chem. Soc.*, 2023, **145**, 19877–19884.

16 S. Liu, C. Zhu, J. Xu, L. Lu, Q. Fang, C. Xu, Y. Zheng, S. Song and Y. Shen, *Appl. Catal. B Environ.*, 2024, **344**, 123629.

17 C. Wu, Z. Teng, C. Yang, F. Chen, H. B. Yang, L. Wang, H. Xu, B. Liu, G. Zheng and Q. Han, *Adv. Mater.*,

2022, **34**, 2110266.

18 Q. Zhi, W. Liu, R. Jiang, X. Zhan, Y. Jin, X. Chen, X. Yang, K. Wang, W. Cao, D. Qi and J. Jiang, *J. Am. Chem. Soc.*, 2022, **144**, 21328–21336.

19 D. Chen, W. Chen, Y. Wu, L. Wang, X. Wu, H. Xu and L. Chen, *Angew. Chem. Int. Ed.*, 2023, **62**, e202217479.
